# Chrom-seq identifies RNAs at chromatin marks

**DOI:** 10.1126/sciadv.adn1397

**Published:** 2024-07-31

**Authors:** Ligang Fan, Wei Sun, Yitong Lyu, Furong Ju, Wenju Sun, Jie Chen, Haiqian Ma, Shifei Yang, Xiaomin Zhou, Nan Wu, Wenkai Yi, Erfei Chen, Rodrigo Villaseñor, Tuncay Baubec, Jian Yan

**Affiliations:** ^1^ Ministry of Education Key Laboratory of Resource Biology and Biotechnology in Western China; Shaanxi Provincial Key Laboratory of Biotechnology; School of Medicine, Northwest University, Xi’an, China.; ^2^ Department of Biomedical Sciences, Jockey Club College of Veterinary Medicine and Life Sciences; The Tung Biomedical Sciences Centre, City University of Hong Kong, Hong Kong SAR, China.; ^3^ Ming Wai Lau Centre for Reparative Medicine, Karolinska Institutet, Hong Kong SAR, China.; ^4^ Division of Molecular Biology, Biomedical Center (BMC), Faculty of Medicine, Ludwig-Maximilians-Universität München, Munich, Germany.; ^5^ Institute of Biodynamics and Biocomplexity, Department of Biology, Science Faculty, Utrecht University, Utrecht, the Netherlands.; ^6^ Department of Precision Diagnostic and Therapeutic Technology, The City University of Hong Kong Shenzhen Futian Research Institute, Shenzhen, China.

## Abstract

Chromatin marks are associated with transcriptional regulatory activities. However, very few lncRNAs have been characterized with the role in regulating epigenetic marks, largely due to the technical difficulty in identifying chromatin-associating RNA. Current methods are largely limited by the availability of ChIP-grade antibody and the crosslinking, which generates high noise. Here, we developed a method termed Chrom-seq to efficiently capture RNAs associated with various chromatin marks in living cells. Chrom-seq jointly applies highly specific chromatin mark reader with APEX2, which catalyzes the oxidation of biotin-aniline to label the adjacent RNAs for isolation by streptavidin-coated beads. Using the readers of mCBX7/dPC, mCBX1, and mTAF3, we detected RNA species significantly associated with H3K27me3, H3K9me3, and H3K4me3, respectively. We demonstrated that Chrom-seq outperformed other equivalent methods in terms of sensitivity, efficiency, and cost of practice. It provides an antibody-free approach to systematically map RNAs at chromatin marks with potential regulatory roles in epigenetic events.

## Teaser

Chrom-seq enables identification of RNA transcripts associated with various chromatin epigenetic modifications.

## INTRODUCTION

A key mechanism in determining the cellular function and plasticity is the regulation of gene transcription. It is widely recognized that all information required for dictating the transcriptional regulation is embedded in the genome. This information comprises both sequence-based genetic factors and nonsequence-based epigenetic features including histone modifications, DNA methylation, chromatin remodeling, RNA processing and modifications, etc. Histone is the major components of chromatin, which serves as the instructive DNA scaffold to regulate the uses of DNA such as replication and transcription. Histone posttranslational modifications (PTMs) encompassing lysine methylation and acetylation have been found highly correlated with transcriptional activity. Deposition and removal of PTMs are catalyzed by highly specific enzymes. For example, histone H3 lysine 4 monomethylation (H3K4me1) and H3 lysine 27 acetylation (H3K27ac) are strongly enriched at active gene distal enhancers, possibly functional in mediating chromatin long-range interaction and binding of transcription factors (*1*, *2*), and are deposited by histone lysine methyltransferases (KMTs) MLL3/4 and histone lysine acetyltransferases (HATs) CBP/p300, respectively. The precise deposition and the removal of histone modifications are critical for cellular fitness and its response to the external cues, related to organismal development and progression of diseases.

Nevertheless, how epigenetic modifications are regulated remains largely elusive. A growing body of evidence indicates that noncoding RNA (ncRNA) plays an important role of epigenetic regulator. In addition to microRNAs (miRNAs) that directly target and silence genes coding for protein complexes and enzymes participating in chromatin epigenetic modification (*3*, *4*), the large class of long ncRNAs (lncRNAs) can serve as signals for decoding chromatin epigenetic modifications. The lncRNAs can interact with modification enzymes, referred to as histone writers, readers, and erasers, hence facilitating the recruitment of them to certain genetic loci for site-specific transcriptional regulation (*5*–*7*). The aberrant expression of these lncRNAs usually leads to severe diseases. For instance, lncRNA ST3Gal6-AS1 interacts with MLL1 and facilitates deposition of the H3K4me3 signal at ST3Gal6 promoter. During colorectal cancer development, expression of lncRNA ST3Gal6-AS1 and ST3Gal6 is dysregulated, which consequently activates phosphatidylinositol 3-kinase (PI3K)/Akt signaling pathway for driving tumor cell proliferation (*8*). Although it becomes clear that lncRNA is one of the general epigenetic regulators, the number of lncRNAs that are identified and functionally characterized only takes up a tiny portion of the known lncRNAs in our genome (*9*), given that the ENCODE database estimated 172,112 human lncRNA species (*10*). The slow progress of finding more epigenetic regulatory lncRNAs impedes the application of using them as diagnostic or prognostic biomarkers and therapeutic targets.

The currently available methods to interrogate the ncRNAs associated with specific chromatin modifications primarily include Chromatin isolation by RNA purification followed by next-generation sequencin (ChRIP-seq) (*11*), Chromatin-associated RNA immunoprecipitation followed by next-generation sequencing (CARIP-seq) (*12*), Profiling interacting RNAs on chromatin followed by deep sequencing (PIRCh-seq) (*13*), and RT&Tag (*14*). Apart from RT&Tag, the former three of the aforementioned methods share a highly similar principle with minor difference mainly in the means of crosslinking. CARIP used 1% formaldehyde for crosslinking RNA-chromatin, but the reaction only lasted for 1 min, namely, that milder crosslinking was preferred. Thus, the inventor reduced the number of sonication cycles, which they claimed that this modification could preserve more RNA-protein interactions. However, ChRIP-seq applied additional ultraviolet (UV) crosslinking to cells following the 10-min treatment of 1% formaldehyde, in combination of intensive sonication (*15*). Obviously, the inventors of ChRIP-seq believed that double crosslinking would better retain RNA-chromatin interactions, although the impact of crosslinking was not systematically discussed. The inventors of PIRCh-seq were apparently aware of the potential issue of crosslinking and thus had tried different crosslinkers. They ultimately chose 1% glutaraldehyde in PIRCh-seq to replace formaldehyde in crosslinking RNA-chromatin, claiming that it could better capture RNA-chromatin association (*13*). Because of the small number of following studies using these methods, the best crosslinking strategy remained inconclusive, and thus, it is best to develop a method that can avoid the crosslinking step. In addition to crosslinking, another major caveat for all of these methods is the dependency on antibody of high quality, usually validated as chromatin immunoprecipitation (ChIP)–grade. Although the histone PTM specific antibodies are the standard reagents in many research laboratories, it has been well recognized that validation of commercial antibodies is not always rigorous, and thus, the lot-to-lot variability of specificity and binding affinity could confer strong influence on the reliability of the scientific results (*16*). Therefore, to pledge the reliability of the data, the ENCODE consortium demands multiple supporting characterizations, such as dot blot assays, immunoblots, and even mass spectrometry for each antibody lot used in generating the published data (www.encodeproject.org/help/antibody_characterization_process/). To fundamentally circumvent the issues of crosslinking and antibody variability, a novel method with alternative solutions is urgently needed.

## RESULTS

To this end, we were motivated to develop Chrom-seq, a method free of both crosslinking and antibody, to identify RNAs associated with epigenetically modified chromatin. The method was based on proximity biotinylation of nucleic acids through jointly applying the epigenetic reader module proteins with APEX2 to living cells. APEX2, an engineered ascorbate peroxidase, could catalyze the one-electron oxidation of biotin-phenol, a membrane permeable small molecule, to generate short-lived (<1 ms) highly reactive radicals that covalently conjugate onto protein or nucleic acids (*17*). It has been revealed that biotin-aniline (Btn-An) is another substrate of APEX2 but with higher reactivity than biotin-phenol in labeling RNA molecules on the presence of H_2_O_2_ (*18*). So, we determined to use Btn-An as the labeling compound in our method. The epigenetic reader modules are all derived from natural proteins, which are evolutionarily conserved, and have been well validated in previous studies, thus having high affinity and specificity (*16*, *19*). The currently well-established reader modules include chromodomain from CBX7 and *Drosophila* Polycomb (dPC) for H3K27me3 (trimethyl-histone H3 lysine 27), chromodomain from CBX1 for H3K9me3 (trimethyl-histone H3 lysine 9), Plant homeodomain (PHD) from TAF3 for H3K4me3 (data file S1), and the methyl-CpG–binding domain (MBD) from MBD1 and MeCP2 for DNA methylation (mostly are methyl-cytosine at CpG motif; not used in this study) (*19*). To guard the specificity of Chrom-seq, we also verified the association of CBX7 with the epigenetically modified genomic loci (fig. S1A).

**Fig. 1.**
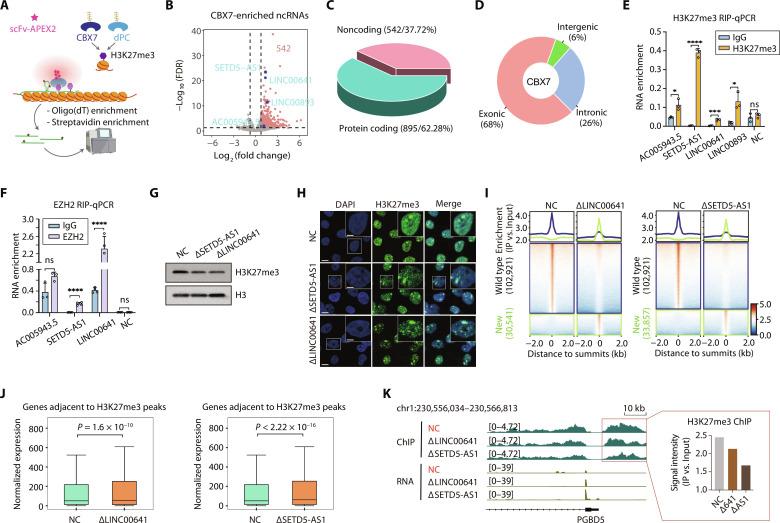
Identification of RNAs associated with H3K27me3 with Chrom-seq. (**A**) Schematic description of the Chrom-seq. Reader (CBX7 and dPC) is used to recognize H3K27me3-modified chromatin. Black stars indicate Btn-An. (**B**) Volcano plot shows the enrichment of ncRNAs in HEK293T cells. Enriched ncRNAs are labeled in red dots (*n* = 542), and the validated RNAs are highlighted in blue. (**C**) 3D pie chart shows the number and percentage of coding and noncoding genes. (**D**) Donut chart shows the proportion of Chrom-seq reads aligned to feature regions classified as exonic, intronic, and intergenic. (**E** and **F**) Validation of candidate genes by RIP-qPCR. RNAs were enriched using H3K27me3 (E) and EZH2 (F) antibodies, respectively. NC, negative control. Statistic *P* values by two-tailed Student’s *t* test are shown. *P < 0.05, ***P < 0.001, and ****P < 0.0001. ns, not significant. (**G**) Western blot of H3K27me3 and H3 in total protein from cells depleted of SETD5-AS1 (∆SETD5-AS1) and LINC00641 (∆LINC00641) using ASOs. (**H**) Confocal fluorescent imaging shows the distribution of H3K27me3 in nucleus (DAPI) in ∆SETD5-AS1, ∆LINC00641, and ASO control (NC) cells. Scale bar on the lower left corner represents a length of 10 μm and 5 μm in the white box inset. (**I**) Profile plots (upper) and heatmaps (lower) represent ChIP-seq signals upon ∆LINC00641 (left) and ∆SETD5-AS1 (right) around wild-type H3K27me3 peaks. (**J**) Box plots show the change of gene expression levels within the ±50 kb range of the H3K27me3 peaks upon ∆LINC00641 (left) and ∆SETD5-AS1 (right). Wilcoxon signed-rank test was performed. (**K**) Genome browser shots show the distribution of ChIP-seq (green) and RNA sequencing (RNA-seq) (yellow) signals from samples of NC, ∆LINC00641, and ∆SETD5-AS1. *PGBD5* gene locus was used as an example. The bar plot shows the quantification of ChIP-seq signal in the designated red box.

To enhance the labeling efficiency, we used a system called SunTag (*20*), in which the epigenetic reader module was fused with 10 copies of SunTag epitopes recognized by the single-chain Fv (scFv) with known sequence. Instead of directly fusing APEX2 to the reader, scFv could bring up to 10 APEX2 molecules to each piece of reader protein, markedly augmenting the focal concentration of the APEX2 enzyme (Fig. 1A). Given the high concentration and enzymatic activity of APEX2, the duration of efficient RNA labeling only took less than 1 min. After Btn-An labeling, the RNA was extracted and purified, and the biotinylated-RNA was subsequently enriched with streptavidin-coated beads. To further mitigate the noise from abundant ribosomal RNAs, polyadenylated [poly (A)^+^] RNA was enriched and primers with randomized sequences were used in the first strand synthesis of cDNA by reverse transcription, followed by template switching and polymerase chain reaction (PCR) amplification to generate the double-stranded DNA library for high throughput sequencing. To call the significantly enriched RNAs, reader-free 10×SunTag was introduced with scFv-APEX2 to cells as control, carried out in parallel with the experiment. We showed that the expression of Chrom-seq components and the introduction of Btn-An did not overtly affect the gene expression pattern in cells (fig. S1B).

As a proof of concept, we first focused on the modification of H3K27me3, one of the most studied epigenetic modifications enriched in repressive regulatory elements. Two distinct reader modules had been developed for ChromID (*19*), chromodomain in mouse CBX7 protein (CBX7 hereafter; amino acids 2 to 76) and dPC protein (dPC hereafter; amino acids 2 to 100) (data file S1). First of all, both reader modules were revealed with high specificity to capture H3K27me3 modified histone in cells, with CBX7 showing higher affinity than dPC (fig. S1C). Then, we aimed to identify RNA species associated with the modified chromatin. By applying Chrom-seq with the CBX7 module, we identified 1437 different RNA species significantly associated with H3K27me3 (log_2_-transformed fold change ≥ 0.75, and false discovery rate (FDR) < 0.05; fig. S1D and data file S2), including 542 ncRNAs (taking up to ~38% of all associated RNAs) (Fig. 1B). When calling significantly associated RNAs, we ensured that sufficient reads were obtained for each sequencing library, showing that more than 80% of the Chrom-seq identified highly confident RNAs could be recovered with ~8 million reads (fig. S1E and data file S3). Next, we examined the list of the 542 H3K27me3-associated lncRNAs and found that many of the previously known regulatory RNAs of H3K27me3 were identified by Chrom-seq, such as H19 (*21*), Nuclear Paraspeckle Assembly Transcript 1 (NEAT1) (*22*), Metastasis Associated Lung Adenocarcinoma Transcript 1 (MALAT1) (*23*), Terminal differentiation-Induced Non-Coding RNA (TINCR) (*24*), and SRY-box transcription factor 2 overlapping transcript (SOX2-OT) (*25*), endorsing the validity of Chrom-seq. Intriguingly, we identified a substantial portion of coding mRNAs also associated with the epigenetic mark (Fig. 1C). Consistently, multiple previous studies have suggested that many mRNAs could function both as templates for protein translation and as RNA enzymes to catalyze biochemical reactions (*26*–*28*). For example, it was reported that >500 mRNAs were revealed interacting with the polycomb repressive complex 2 (PRC2) complex, the specific writer enzyme for H3K27me3 in mammalian cells (*28*, *29*), therefore indicating the dual role of these mRNAs in regulating chromatin epigenetic modifications besides protein coding (*30*). To exclude the possibility that the capture of these RNAs was caused by their high abundance in cells, we showed that the CBX7 Chrom-seq–enriched RNAs displayed significantly lower expression level than the overall distribution of the cellular transcriptome (Mann-Whitney *U* test, *P* < 2.2 × 10^−16^; fig. S1F).

In contrast, dPC only found 702 RNAs significantly associated with H3K27me3 (fig. S2A and data file S2), of which 241 were noncoding (~34% of all associated RNAs, similar to CBX7) (fig. S2, B and C). In both experiments, the mapped reads of Chrom-seq libraries were primarily aligned to exons (68 and 69% for CBX7 and dPC, respectively), with a small fraction of the intronic (26 and 26%) and intergenic (6 and 5%) origins (Fig. 1D and fig. S2D), demonstrating that the signal of Chrom-seq was exclusively from matured RNA instead of genomic DNA contamination, an important indicator of the success of RNA capture. The high portion of matured RNAs in capture preserved regulatory interactions in trans between RNAs and chromatin. When comparing the Chrom-seq results from CBX7 and dPC, we found that CBX7 could capture most of and remarkably more RNA species found by dPC, these including both coding and noncoding transcripts (fig. S2E). The smaller number of captures by dPC than CBX7 was unlikely caused by the cellular abundance of RNAs (fig. S2F) but could be due to its lower affinity to some H3K27me3 loci revealed by the pulldown experiment (fig. S1C). Therefore, we mostly focused on CBX7 data in the subsequent analyses.

To investigate the involvement of ncRNAs in the regulation of epigenetic modifications, we selected several lncRNAs with moderate enrichment levels for validation. These include AC005943.5, SETD5-AS1, LINC00641, and LINC00893 (Fig. 1B). We confirmed the interaction between these lncRNAs and H3K27me3-modified chromatin with RNA immunoprecipitation quantitative PCR (RIP-qPCR). The abundantly expressed lncRNA BCYRN1 was used as negative control. As expected, all of them displayed significant association except the negative control (Fig. 1E). In mammalian cells, deposition of H3K27me3 onto chromatin is mainly catalyzed by the conserved PRC2, comprising the EZH1/2 catalytic subunit, SUZ12, EED, and RBBP7/4 (*29*). Next, we asked whether these lncRNAs played a role in facilitating the recruitment of H3K27me3-specific methyltransferase. By performing RIP using antibody specifically against the EZH2 catalytic subunit, two candidates, SETD5-AS1 and LINC00641, exhibited significant enrichment in the EZH2 pulldown RNAs (Fig. 1F). To further assess the role of these lncRNAs in other cell types, we performed RIP in HCT116 cells, a human colon cancer cell line, using the H3K27me3 and EZH2 antibodies, respectively. The qPCR results confirmed the association of SETD5-AS1 and LINC00641 to H3K27me3 (fig. S3A) and EZH2 (fig. S3B), suggesting the conserved function of lncRNA across different cell types.

To unravel the regulatory role of both lncRNAs, we knocked down the RNA using antisense oligonucleotides (ASOs) (fig. S3C). A slight but significant reduction of H3K27me3 Western blotting (WB) signal was observed in cells deficient of either SETD5-AS1 or LINC00641 (Fig. 1G). Consistently, we also observed diminished immunofluorescent signals reflecting levels of H3K27me3 in knockdown cells compared to mock control (Fig. 1H and fig. S3D).

Subsequently, we examined whether the decrease in epigenetic modification occurred globally or only in certain specific loci that were more significantly affected than others. Therefore, ChIP-seq of H3K27me3 was performed. Most of the H3K27me3 peaks in wild-type cells exhibited a significant reduction of ChIP-seq signal when SETD5-AS1 or LINC00641 was depleted (Fig. 1I), revealing the impact of these lncRNAs on preserving the H3K27me3 signal in the genome. As expected, this epigenetic change was accompanied by significant up-regulation of genes located adjacent to these affected regions (Fig. 1J). Intriguingly, besides the overall decline in H3K27me3 levels across the wild-type peaks, we also observed the emergence of new H3K27me3 peaks at a number of loci (Fig. 1I). This phenomenon could potentially be attributed to an indirect influence of the lncRNA function, which required additional investigations to gain a comprehensive understanding of the underlying regulatory mechanism. The result suggests that these lncRNAs are essential for maintaining the epigenetic landscape of H3K27me3 in the human genome. To better illustrate the change, a genome browser snapshot around the *PGBD5* gene locus was taken for example, showing that H3K27me3 signal around the gene *PGBD5* promoter was notably reduced upon depletion of SETD5-AS1 or LINC00641. Meanwhile, the *PGBD5* gene was activated (Fig. 1K).

It had been reported that H3K27me3-associated RNAs were mostly transcribed from silenced genomic loci (*14*), consistent with our observation that CBX7-captured RNAs showed significantly lower expression level than other genes (fig. S1F). When specifically examining the H3K27me3 and H3K9me3 ChIP-seq signals, we found that H3K27me3-associated transcripts were indeed derived from regions decorated with higher H3K27me3 and H3K9me3 ChIP-seq signals but lower active H3K4me3 ChIP-seq signal at their transcription starting site and gene bodies than nonenriched transcripts (fig. S3E). These results revealed that the association of repressive chromatin regions with transcripts suggests a nonlocal regulatory role of these RNAs. For example, XIST RNA could travel remotely to bring the PRC2 complex to various zones spreading the entire X chromosome for deposition of H3K27me3 and inactivation of transcription (*31*).

**Fig. 2.**
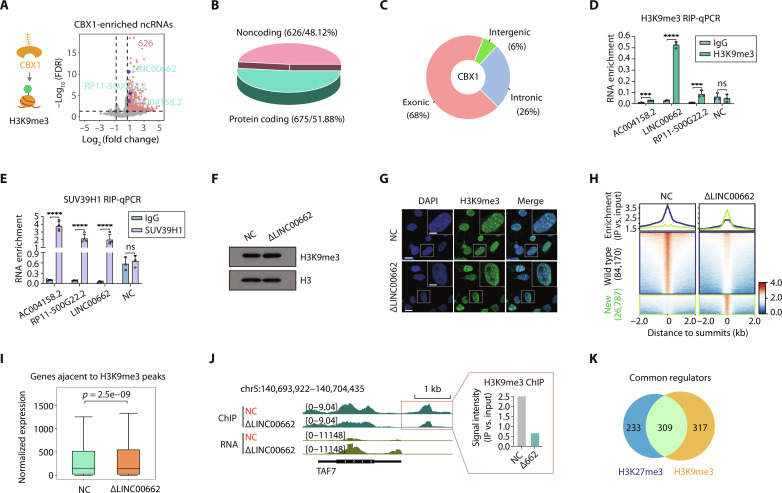
RNAs associated with H3K9me3. (**A**) RNAs were enriched by the CBX1 reader in HEK293T cells. Left: Scheme of CBX1 Chrom-seq. Brown crossed lines indicate 10×GCN4 peptides. Right: Volcano plot shows the enrichment of ncRNAs associated with H3K9me3 modification. Enriched ncRNAs are labeled in red (*n* = 626), and the validated RNAs are highlighted in blue. (**B**) 3D pie chart shows the number and percentage of coding and noncoding genes. (**C**) Donut chart shows the proportion of Chrom-seq reads aligned to feature regions classified as exonic, intronic, and intergenic. (**D** and **E**) Validation of candidate genes by RIP-qPCR. RNAs were enriched using H3K9me3 (D) and SUV39H1 (E) antibodies, respectively. Statistic *P* values by two-tailed Student’s *t* test are shown. ***P < 0.001 and ****P < 0.0001. (**F**) Western blot of H3K9me3 and H3 in total protein from cells depleted of the LINC00662 (∆LINC00662) using ASOs. NC ASO that does not target any sequence. (**G**) Confocal fluorescent imaging shows the distribution of H3K9me3 in nucleus (DAPI) in ∆LINC00662 and ASO control (NC) cells. Scale bars on the lower left corner represent a length of 10 μm and 5 μm in the white box inset. (**H**) Profile plots (top) and heatmaps (bottom) represent ChIP-seq signals upon ∆LINC00662 around H3K9me3 peaks. (**I**) Box plots show the change of gene expression levels within the ±50 kb range of the H3K9me3 peaks upon ∆LINC00662. Wilcoxon signed-rank test was performed. (**J**) Genome browser shots show the distribution of ChIP-seq (green) and RNA-seq (yellow) signals from samples of NC and ∆LINC00662. *TAF7* gene locus was used as an example. The bar plot shows the quantification of ChIP-seq signal in the designated red box. (**K**) Venn diagram shows common ncRNA regulators of H3K27me3 and H3K9me3.

Inspired by the results, we applied Chrom-seq to H3K9me3 using the reader component of CBX1 chromodomain (CBX1 hereafter; amino acids 18 to 82) (Fig. 2A and data file S1). First, we confirmed the affinity of CBX1 to H3K9me3-decorated chromatin by immunoprecipitation (fig. S4A). The result of Chrom-seq showed that 1301 RNA species were significantly associated with H3K9me3 (log_2_-transformed fold change ≥ 0.75 and FDR < 0.05; fig. S4B and data file S4), including 626 ncRNAs (Fig. 2B). Similar to H3K27m3 Chrom-seq, CBX1-captured reads were also mostly derived from annotated exons (68%), excluding the DNA contamination (Fig. 2C). Besides the known regulators of H19 (*32*) and MALAT1 (*33*), we confirmed the interaction of a few novel lncRNAs, such as AC004158.2, LINC00662, and RP11-500G22.2 using RIP-qPCR in both human embryonic kidney (HEK) 293T cells (Fig. 2D) and HCT116 cells (fig. S4C). The deposition of H3K9me3 is catalyzed by a family of SET-domain containing methyltransferases, primarily SETDB1 (*34*), SUV39H1, and SUV39H2 (*35*). Thus, we examined the interaction between SUV39H1 and the candidate lncRNAs with RIP-qPCR and found that all of them significantly bound to the SUV39H1 enzyme in both cell types (Fig. 2E and fig. S4D).

We took one of the top enriched candidates LINC00662 for functional characterization. Unlike H3K27me3, when cells were deficient of LINC00662 RNA, we did not observe overt change of H3K9me3 abundance by WB analysis (Fig. 2F). Consistently, we barely detected subtle change at marginal significance with the immunofluorescent imaging assay (Fig. 2G and fig. S4F), suggesting that the impact of this lncRNA was likely to be locus specific. Hence, H3K9me3 ChIP-seq was used, showing a notable reduction of signals around the wild-type peaks when LINC00662 was knocked down (Fig. 2H and fig. S4E). Proximal genes were also consequently up-regulated (Fig. 2I). For example, the enrichment of H3K9me3 at both the promoter and the gene body of *TAF7* was remarkably dampened upon knockdown of LINC00662, consequently boosting the transcription of the nearby *TAF7* gene (Fig. 2J). Analogous to interference against the regulatory lncRNAs of H3K27me3 (Fig. 1I), new H3K9me3 peaks appeared in many loci upon LINC00662 depletion (Fig. 2H), suggesting a role of the lncRNA in maintenance of H3K9me3 landscape.

RNAs associated with H3K9me3 were also mostly transcribed from repressive genomic regions (fig. S4G) and were expressed at a lower level than the overall transcriptome (fig. S4H). When we compared ncRNAs associated with both repressive marks, more than one-third of them (309 of 859) exhibited linkage with both H3K9me3 and H3K27me3 (Fig. 2K). Such a high ratio of overlap could come from multiple sources: On one hand, evidences have emerged that these two repressive marks co-occurred at some developmentally repressed genes and transposable elements (*36*–*38*), and thereby, lncRNAs associated with these bivalent regions were identified; or on the other hand, some lncRNAs were involved in regulation of both epigenetic marks in different loci; furthermore, it had been reported that CBX7 could have some weak affinity toward H3K9me3, especially in cells with low H3K27me3 signals (*39*), so that CBX7 captured some H3K9me3-associated lncRNAs. It is noteworthy that the lncRNAs identified by dPC Chrom-seq, which was revealed with no cross-reactivity with H3K9me3 (*19*), also exhibited significant overlap with CBX1 captures (fig. S4I), providing further support for the hypothesis that these signals likely represent shared regulatory transcripts. In addition, the chromatin-associated proteomes of both marks also substantially overlapped (*19*), together suggesting the possible interplay between these epigenetic modifications in regulating gene silencing.

So far, we have demonstrated the Chrom-seq as a robust and highly efficient method to identify lncRNAs that were associated with various epigenetic marks mediating the transcriptional activity. The major advancement of Chrom-seq compared to the currently available methods to identify RNAs associated with epigenetic modifications—such as PIRCh-seq, ChRIP-seq, CARIP-seq or RT&Tag—is that Chrom-seq no longer depends on antibody capture or covalent cross-linking, both of which are the major sources to cause experimental variation. Besides, the efficiency has also been significantly improved in terms of the number of captured lncRNAs, amount of input material, sequencing depth, and cost (Table 1 and data file S3). In addition, principal components analysis also demonstrated high reproducibility of results from independent replicative experiments (fig. S5A). RT&Tag (*14*), which had been most recently developed, made remarkable advancement beyond other equivalent methods, such as RIP-seq/CLIP-seq (Cross-linking and immunoprecipitation followed by next-generation sequencing) and PIRCh-seq/ChRIP-seq, in many aspects, particularly its broad application, crosslinking-free nature, and low cost. It claimed that as few as ~100,000 cells were needed for one assay, which was more than 50-fold fewer than its counterparts. However, the demonstration of RT&Tag in the study (*14*) had only been applied to the *Drosophila* cell that has a much smaller (>20 times) genome size (120 Mb) than the mammalian cells (1.6 to 6.3 Gb) (*40*). Such a method still demanded the high quality of antibody to recognize specific histone modifications, limiting its applicability.

**Table 1.** Comparison of Chrom-seq with antibody-based methods. The comparison includes the amount of cells used for one experiment (input material), and whether covalent crosslinking (crosslinking), ChIP-grade antibody (antibody), or ultrasonication for chromatin fragmentation (sonication) is needed, the amount of next-generation sequencing reads sufficient for analysis of one experiment (sequencing reads), the duration (time), and cost (in U.S. dollars) of one experiment. Note that “Yes/No” means some applications implemented the action while the others did not.

Methods	Chrom-seq	RT&Tag (*Drosophila* cells)	RIP-seq	PIRch-seq	ChRIP-seq	CARIP-seq
**Input material**	0.5 million	0.1 million	10 million	4 million	20 million	10 million
**Crosslinking**	No	No	Yes/No	Yes	Yes	Yes
**Antibody**	No	Yes	Yes	Yes	Yes	Yes
**Sonication**	No	No	Yes/No	Yes	Yes	Yes
**Sequencing reads**	10–15 million	4–8 million	20 million	20 million	50 million	20 million
**Time**	2 days	1–2 days	3 days	3 days	3 days	3 days
**Cost ($)**	25	50	150	225	200	200

**Fig. 3.**
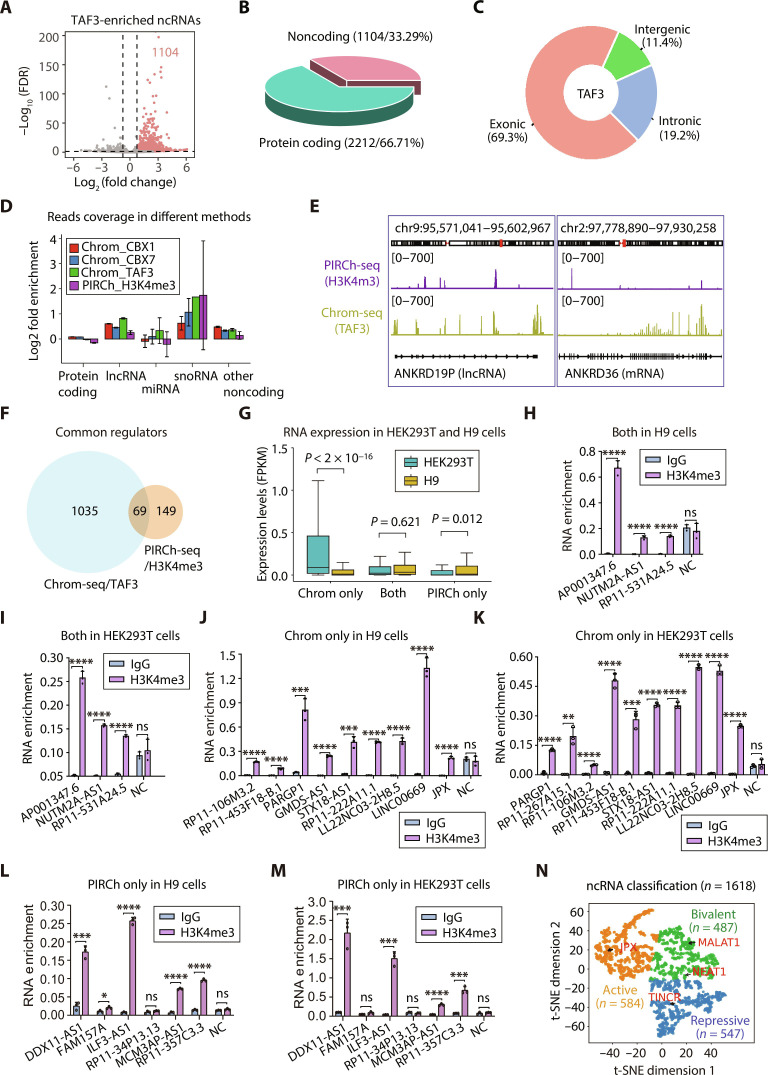
Comparison with PIRCh-seq. (**A**) RNAs enriched by TAF3 reader in HEK293T cells. The volcano plot shows the enrichment of ncRNAs associated with H3K4me3 modification. The number of enriched ncRNAs is 1104. (**B**) 3D pie chart shows the number and percentage of coding and noncoding genes. (**C**) Donut chart shows the proportion of Chrom-seq reads aligned to feature regions classified as exonic, intronic, and intergenic. (**D**) Bar plots show the fold enrichment of the protein coding gene, lncRNA, miRNA, snoRNA, and other ncRNA associated with specific histone modifications (H3K4me3, H3K9me3, and H3K27me3) identified by Chrom-seq in HEK293T or by PIRCh-seq in H9 cells. Error bar indicates the SE. (**E**) Genome browser shots show the distribution of Chrom-seq and PIRCh-seq signals along the gene body of captured RNAs. *ANKRD19P* and *ANKRD36* gene loci are used as examples. (**F**) Venn diagram shows comparison of ncRNAs found by TAF3 Chrom-seq and H3K4me3 antibody-based PIRCh-seq. (**G**) Box plots show the expression levels of RNAs enriched by Chrom-seq and PIRCh-seq in HEK293T and H9 cells, respectively, corresponding to (F). Mann-Whitney *U* test was performed. (**H** to **M**) RIP-qPCR validation of TAF3 Chrom-seq and PIRCh-seq enriched genes in H9 [(H), (J), and (L)] and HEK293T [(I), (K), and (M)] cells, respectively, including genes found by both Chrom-seq and PIRCh-seq [(H) and (I)], those only found by Chrom-seq [(J) and (K)], and those only found by PIRCh-seq [(L) and (M)]. Statistic *P* values by two-tailed Student’s *t* test are shown. *P < 0.05, **P < 0.01, ***P < 0.001, and ****P < 0.0001. IgG, immunoglobulin G. (**N**) Classification of ncRNAs according to their association with different chromatin marks in HEK293T cells. Scatter plots show the t-SNE visualization of ncRNA K-means clustering based on their fold enrichment in Chrom-seq.

To ensure an equitable comparison of applicability, we selected PIRCh-seq for a more focused analysis, given the previous demonstration indicating superior performance of PIRCh-seq over other existing methodologies in mammalian cells (*13*). Because only H3K4me3 data were available for published PIRCh-seq dataset performed in human cells, we performed Chrom-seq using the PHD domain of mouse TAF3 (TAF3 hereafter; amino acids 841 to 932; data file S1) (*19*) to target histone H3 lysine 4 trimethylation (H3K4me3), an epigenetic marker of the active promoter in HEK293T cells (Fig. 3A). As a result, Chrom-seq identified 3316 different RNA species in HEK293T cells (log_2_-transformed fold change ≥ 0.75 and FDR < 0.05; fig. S5B and data file S5), including 1104 ncRNAs (Fig. 3B). Likewise, the reads were mostly aligned to human exons (69.3%; Fig. 3C), verifying their derivation from RNA transcript. Meanwhile, we obtained the PIRCh-seq data of H3K4me3-enriched RNAs in a human embryonic stem cell line H9 (*13*).

We first compared the reads coverage showing that Chrom-seq using different reader modules all exhibited significantly higher signal enrichment particularly in lncRNAs and other ncRNAs (Fig. 3D), illustrated in the ANKRD19P and ANKRD36 loci (Fig. 3E). Next, we compared the H3K4me3-associated RNAs identified by the two methods. On the basis of the original publication, the PIRCh-seq used a very relaxed cutoff (log_2_-transformed fold change > 0 and *P* < 0.05) to define the significantly enriched lncRNA. Even so, only 218 ncRNAs were detected, notably fewer than what Chrom-seq identified with a more stringent cutoff (1104 lncRNA species under the cutoff log_2_-transformed fold change ≥ 0.75 and FDR < 0.05). Although the two experiments were conducted in two different cells, Chrom-seq recovered one-third of ncRNAs (69 of 218) found by PIRCh-seq in H9 human embryonic stem cell line (Fig. 3F). The failure of Chrom-seq in finding the remaining 149 H3K4me3-associated ncRNAs could be due to the cell type–specific expression of these RNAs. The 149 ncRNAs specifically identified by PIRCh-seq (annotated as “PIRCh only”) exhibited significantly lower expression in HEK293T cells compared to their abundance in H9 cells (Fig. 3G). Meanwhile, the expression level of ncRNAs unique to Chrom-seq but missing from PIRCh-seq (annotated as “Chrom only”) showed significantly higher expression in HEK293T cells (Fig. 3G).

We further tested some of the interactions in both HEK293T and H9 cells with an orthogonal method, RIP-qPCR. First, we confirmed the H3K4me3-associated lncRNAs found by both methods. As expected, all of them exhibited significant interactions in both cell types (Fig. 3, H and I). Then, we selected nine lncRNAs from the 1035 Chrom-only candidates with high expression in both HEK293T and H9 cells for validation. The RIP-qPCR reaffirmed that these lncRNAs engaged in interactions with H3K4me3 across both cellular contexts, thereby indicating that the PIRCh-seq may have failed to capture these molecular associations (Fig. 3, J and K). On the other hand, we selected six lncRNA candidates from the 149 “PIRCh-only” group, exhibiting moderate to high expression levels in both cell lines, to perform RIP-qPCR. The experimental results revealed that five of these lncRNAs exhibited significant binding to the H3K4me3 histone mark in the H9 cell context (Fig. 3L), of which 4 were also found to be enriched by H3K4me3 in HEK293T cells (Fig. 3M). These findings suggested that PIRCh-seq was able to identify certain lncRNA species that the Chrom-seq method had failed to detect (Fig. 3, L and M), endorsing the complementary nature of the two methodologies. Notably, the moderately expressed lncRNA RP1-34P13.13 in both cell types failed to be validated by RIP-seq, thereby indicating the presence of some false-positive signals generated by the PIRCh-seq technique. Together, the difference shown in Fig. 3F is caused by both the cell type–specific binding and technical variation. Overall, Chrom-seq outperformed existing methods in detecting lncRNA associated with chromatin epigenetic modifications, in addition to its independency of antibody or covalent crosslinking.

## DISCUSSION

So far, we have developed a novel method Chrom-seq to identify RNAs associated with various histone PTMs, both active and repressive, and validated the molecular function of some identified RNAs. We have noticed that some lncRNAs can be associated with multiple different PTMs (Fig. 2K). To further explore the function of the chromatin-associated RNAs, we used *K*-means clustering to classify them based on the type of epigenetic marks, which they were associated with (see Materials and Methods for details). We found that some lncRNAs could even be associated with both active and repressive PTMs, defined as bivalent chromatin-associated RNAs, while others could only interact with one type of PTMs, either active chromatin-associated RNAs or repressive chromatin-associated RNAs (Fig. 3N, fig. S5C, and data file S6). The comprehensive elucidation of the biological functions of these RNAs warrants further investigation in subsequent studies.

Outperforming the currently available methods, such as RIP-seq/CLIP-seq, PIRCh-seq/ChRIP-seq, and even the most recently developed RT&Tag, the Chrom-seq method demanded remarkably lower amount of cells, sequencing reads, and thus strikingly lower cost (Table 1). In addition, our method occurs under near-physiological conditions and avoids small molecule or UV-mediated crosslinking, markedly reducing the nonspecific interactions. ChIP-grade antibody is no longer required, replaced by the physiological reader domains of the targeted marks. On the one hand, this could avoid the large lot-to-lot quality variation of antibody that could lead to strong experimental noise, warned by ENCODE consortium and other scientists (*16*, *41*, *42*). On the other hand, the naturally occurring and engineered reader protein exceeds the antibody in terms of robustness, sensitivity, and specificity, given the million years of evolution for precise control of epigenetic modifications and gene transcription (*16*). We showed that Chrom-seq identified >20 times the number of ncRNAs associated with the same H3K4me3 PTM than PIRCh-seq, caused by low efficiency of either crosslinking or antibody recognition (Fig. 3). Other advantages include that: (i) protein engineering of the reading domains would allow for the generation of higher affinity and specificity, recognition of novel modification such as *N*^6^-methyladenosine (m^6^A) or pseudouridine (Ψ), joint recognition of multiple different domains in one reaction, e.g., bivalent domains, and addition of fluorescent or affinity tags for simultaneous visualization and (ii) lower cost reduced from purchasing ChIP-grade antibody and sequencing makes it feasible for scaling up the analyses in complicated biological systems. We can investigate the regulatory RNAs of various PTMs in a spectrum of tissues in an organism or in cells under different circumstances, as long as they are transfecting host. For example, we applied Chrom-seq to cells of different origins—such as HCT116, NIH3T3, and Neuro-2a—and successfully identified many regulatory lncRNAs in the mouse genome (data file S5).

We also noticed some limitations of Chrom-seq. The Chrom-seq component should be transfected and expressed into the living cells. First, it could be easily applied to transfecting host cells but has difficulty dealing with cells hard to transfect. To address this issue, we are further developing Chrom-seq compatible with protein- or mRNA-mediated delivery of the engineered chromatin reader–APEX components, expecting to broaden its applicability. In addition, the overexpression of Chrom-seq component is also likely to induce certain alteration of cells (observer effect), although we did not detect any overt transcriptional change (fig. S1B). This could still cause the disruption of the interactions between targeted chromatin loci and adjacent RNAs. Furthermore, because the oxidized Btn-An is highly reactive with guanosine, RNA species with more G nucleotide exposed in nonstructural regions could confer bias in APEX2 labeling. These technical variations might partially explain the failure of capturing some lncRNAs (Fig. 3M). Therefore, Chrom-seq and the existing methods could be mutually complementary means to comprehensively find the chromatin-associated RNAs.

For future applications, epigenetic modifications in DNA or RNA could also be targeted using specific reader modules. For example, methylation at carbon 5 within CpG motif of genomic DNA could be recognized by MBD proteins, such as MeCP2, MBD1, etc., which had been established in the ChromID study (*19*). Interest has risen in understanding the function of epigenetic modifications in various RNA species, particularly the abundant ones, i.a., m^6^A (*43*) or pseudouridine (Ψ) (*44*). Specific reader proteins are known, namely, YTHDC1 for m^6^A and the yeast Prp5 RNA helicase for Ψ. Application of ChromID and Chrom-seq to these modifications will no doubt improve the knowledge and visibility of these epigenetic codons, ultimately to refine the central dogma of molecular biology.

## MATERIALS AND METHODS

### Cell culture

HEK293T cells were cultured in the Dulbecco’s modified Eagle’s medium (DMEM; Gibco, 11995040) medium with 10% fetal bovine serum (FBS; Gibco, 10270106). HCT116 cells were cultured in the McCoy’s 5A modified medium (Gibco, 16600082) with 10% FBS. The human embryonic stem cells (H9) were cultured in mTeSRTM1 basal medium (STEMCELL Technologies, 85851) supplemented with mTeSRTM1 supplement (STEMCELL Technologies, 85852). All the culture dishes were coated with Corning Matrigel Matrix (354277) diluted in DMEM/F-12 (Thermo Fisher Scientific, 11330032) at room temperature at least 1 hour before use. In addition, the Gentle Cell Dissociation Reagent (STEMCELL Technologies, 100-0485) was used during H9 cell passage and harvest for cell digestion at 37°C for 3 min. After discarding the supernatant, cells were scraped off and resuspended in the complete mTeSRTM1 medium for downstream usage. A final concentration of 10 μM of Y-27632 (STEMCELL Technologies, 72304) was added to the medium at each passage to inhibit H9 cell differentiation. All cells were cultured at 37°C under 5% CO_2_.

### Plasmid construction

To obtain ACT-scFv-APEX2-P2A-EGFP (enhanced green fluorescent protein) plasmid, the chicken β-actin promoter, scFv and APEX2 fragments were amplified from the ACT-scFv-sfGFP-FokI (our laboratory) and mito-V5-APEX2 (Addgene, 72480) plasmids as templates using the primers in data file S1, respectively. Then, three fragments were cloned into pcDNA3.1-P2A-EGFP vector using the recombinase (ClonExpress Ultra One Step Cloning Kit, Vazyme Biotech, C115-02), fused to the N termini of EGFP immediately following the chicken β-actin promoter. To clone pcDNA3.1-readers-10×GCN4-P2A-mCherry plasmids, we first synthesize the 10×GCN4-P2A-mCherry fragment and insert it into pcDNA3.1 vector to generate pcDNA3.1-10×GCN4-P2A-mCherry vector using the primers in data file S1. Then, CBX7, dPC, CBX1, and TAF3 reader modules were generated by PCR using the templates including CBX7-BASU, dPC-BASU, CBX1-BASU, and TAF3-BASU from T.B.’s laboratory (*19*) using the primers in data file S1, respectively. Then, the resultant PCR products were constructed into pcDNA3.1-10×GCN4-P2A-mCherry plasmid using the recombinase (ClonExpress Ultra One Step Cloning Kit, Vazyme Biotech, C115-02), fused to the N termini of mCherry, following the cytomegalovirus promoter.

### Chrom-seq

HEK293T cells were seeded in the 12-well plates. Both ACT-scFv-APEX2-P2A-EGFP and pcDNA3.1-readers (CBX7, dPC, CBX1, and TAF3)-10×GCN4-P2A-mCherry plasmids were transfected into the HEK293T cells at 80% confluency to coexpress the reader-GCN4 and scFv-APEX2 fusion protein. Reader-free empty vector was used for a negative control. After incubating for 48 hours, fresh medium was changed supplemented with 500 μM Btn-An (Iris Biotech GMBH, LS-3970), and cells were incubated at 37°C under 5% CO_2_ for 30 min. H_2_O_2_ (Anaqua, 7722841) was then added to each well to reach a final concentration of 1 mM with gentle shaking for 1 min. The reaction was quenched by replacing the medium with an equal volume of quenching buffer [5 mM Trolox and 10 mM sodium ascorbate in Dulbecco’s phosphate-buffered saline (DPBS)]. Cells were washed with quenching buffer for three times before proceeding to RNA experiments. One milliliter quenching buffer with 4 μl of Ribolock ribonuclease (RNase) inhibitor (Thermo Fisher Scientific, EO0384) was added to each well. The cells were then scrapped off 12-well plates, transferred to 1.5-ml tubes, and centrifuged at 1000 rpm for 2 min to pellet down cells. The supernatant was then removed, and the total RNA was extracted from cells using the TaKaRa MiniBEST Universal RNA Extraction Kit (TaKaRa, 9767).

The Pierce streptavidin magnetic beads (Thermo Fisher Scientific, 88816) were used to enrich biotinylated RNAs (with an amount of 4 μl of beads per 10 μg of RNA). In general, ~20 μg of RNA was sufficient for generating high-quality Chrom-seq sequencing library. The beads were first washed three times in buffer A [5 mM tris-HCl (pH 7.5), 0.5 mM EDTA, 1 M NaCl, and 0.1% Tween 20 (Sigma-Aldrich, P9416)], followed by two times in buffer B (0.1 M NaOH and 0.05 M NaCl), and one time in buffer C (0.1 M NaCl). Then, beads were resuspended in 150 μl of 0.1 M NaCl and incubated with 150 μl of RNA (diluted in RNase-free water) for 2 hours at 4°C on a rotator. After incubation, the supernatant was removed, and the beads were washed three times in buffer A and resuspended in 54 μl of nuclease-free H_2_O. Subsequently, 33 μl of 3× proteinase buffer (100 μl of buffer contained 30 μl of 10× PBS (pH 7.4) (Invitrogen, AM9625), 30 μl of 20% *N*-Lauryl sarcosine sodium solution (Sigma-Aldrich, L7414), 6 μl of 0.5 M EDTA (Invitrogen, AM9260G), 1.5 μl of 1 M dithiothreitol (DTT; Thermo Fisher Scientific, R0861) and 32.5 μl of water) were respectively added to the beads supplemented with 10 μl of proteinase K (20 mg/ml; Invitrogen, AM2548) and 3 μl of Ribolock RNase inhibitor. The mixture was incubated at 42°C for 1 hour, followed by 55°C for 1 hour on a Thermoshaker (Eppendorf). The enriched RNA was then purified using the “RNA Clean and Concentrator 5” Kit (Zymo Research, R1016). For Chrom-seq library preparation, poly (A)^+^ RNA was selected using the NEBNext Poly(A) mRNA Magnetic Isolation Module (NEB, E7490L) to perform the Smart-seq3 procedure (*45*) with minor modification, including that poly (A)^+^ RNA was fragmented at 85°C for 7 min instead of treating cDNA with the Tn5 and that oligo dT primer was replaced with N6 random primer in the reverse transcription reactions. Briefly, poly (A)^+^ RNA was fragmented at 85°C for 7 min in 5×Maxima H RT buffer [125 mM tris-HCl (pH 8.0), 150 mM NaCl, and 12.5 mM MgCl_2_] with 20 μM N6 random primer. The fragmented RNA was then used to synthesize the first strand of cDNA using the reverse transcriptase (Thermo Fisher Scientific, EP0753) in TS buffer [8 mM DTT, 1 mM deoxynucleotide triphosphate (dNTP), 1 mM guanosine 5′-triphosphate, 2 μM template-switching oligonucleotide (TSO) primer (data file S1), and RNase Inhibitor (100 U/μl)] with a template-switching progress. Immediately, the following procedure was done: 42°C for 90 min with one cycle, 50°C for 2 min, 42°C for 2 min with 10 cycles, and 85°C for 5 min to inactivate the enzyme. Subsequently, cDNA was cleared using the DNA Clean Beads (Vazyme Biotech, N411-02) to generate the DNA library by RCR amplification using the NEBNext Ultra II Q5 Master Mix (NEB, M0544L) and primers (data file S1) for high throughput sequencing. Last, the resultant libraries were purified using the DNA Clean Beads, followed by sequencing using the NovaSeq 6000 S4 PE150 of Illumina platform (Biopharmaceutical Public Service Platform, Nanjing).

### RNA sequencing

For RNA sequencing (RNA-seq) library preparation, total RNA was extracted from cells using the TaKaRa MiniBEST Universal RNA Extraction Kit. Then, the poly (A)^+^ RNA was enriched using the NEBNext Poly(A) mRNA Magnetic Isolation Module to construct the RNA-seq library according to the progress of Chrom-seq library. Subsequently, the resultant libraries were sequenced using the NovaSeq 6000 S4 PE150 of Illumina platform (Biopharmaceutical Public Service Platform, Nanjing).

### ChIP-seq

To validate the function of lncRNA, ASOs including ASOSETD5-AS1, ASOLINC00641, and ASO LINC00662 were transfected into HEK293T cells grown on the six-well plates, respectively. ASOXNC23 was used for the negative control (sequence in data file S1). After 48-hour incubation, 1% formaldehyde (Sigma-Aldrich, F8775) was added into each well to crosslink the cells for 10 min with a slight shaking at room temperature. The crosslink reaction was quenched with ice-cold PBS containing a final concentration of 0.125 M Glycine with a gentle shaking for 5 min. The cells were washed twice with 5 ml of cold PBS and scrapped off the plate. The cells were transferred to 15-ml tubes and pelleted by centrifuging at 500*g* for 5 min. Cells were then resuspended with 10 ml of hypotonic lysis buffer [1 mM EDTA, 10 mM KCl, 20 mM Hepes (pH 7.9), 10% glycerol, and 1 mM DTT, 1× Protease inhibitors (Roche)] for 15 min with a mild shaking and spun down at 500*g* for 5 min at 4°C. For sonication, every 10 million cells were lysed with 1 ml of radioimmunoprecipitation assay (RIPA) buffer [140 mM NaCl, 1 mM EDTA, 1% Triton X-100, 0.1% SDS, 0.1% sodium deoxycholate, 10 mM tris-HCl (pH 8.0), and 1× Proteinase inhibitor (Roche)]. Lysate was then sonicated until the chromatin size at ~300 to 600 base pairs, and the lysate was clear. Covaris M220 sonicator was applied with the following settings: peak power of 75, duty factor of 5%, and cycles/burst of 200, duration for 5 min. The lysates were then spun down at 20,000*g* for 10 min at 4°C. Supernatants were diluted with RIPA buffer, and 50 μl of sample was set aside as an input. The remaining supernatants were incubated with H3K27me3 (Abcam, ab192985) or H3K9me3 (Abcam, ab8898) antibody overnight under rocking in cold room (4°C). On the following day, Pierce Protein A/G Plus Agarose beads (Thermo Fisher Scientific, 20423) that were blocked with RIPA containing 0.5% bovine serum albumin (BSA) were added into the samples followed by incubation for 2 hour in cold room under rocking. The beads were then washed three times with RIPA buffer, followed by two times in RIPA buffer with 0.5 M NaCl, once in LiCl buffer [250 mM LiCl, 1 mM EDTA, 0.5% IGEPAL CA-630, 0.1% sodium deoxycholate, and 10 mM tris-HCl (pH 8.0)], and twice in ice-cold Tris-EDTA (TE) buffer. Each time, the beads were sustaining for 5 min with a gentle rock. After washing, both the beads and input sample were added with 150 μl of extraction buffer [1% SDS in 1× TE, 12 μl of 5 M NaCl, and 10 μg of RNAse A) and incubated at 37°C for 1 hour with a shaking. Next, 20 μg of Proteinase K was added to the samples for incubation at 65°C overnight with thermoshaking. DNA was isolated by performing a phenol/chloroform/isoamyl alcohol extraction to prepare the ChIP-seq library using the NEBNext Ultra II DNA Library Prep Kit for Illumina (NEB, E7645). The library was sequenced using the NovaSeq 6000 S4 PE150 of Illumina platform (Nanjing).

### RNA immunoprecipitation

RIP assay was performed according to previous description by Rinn *et al.* (*46*). The specific process is as follows: 10 million cells were harvested by trypsinization and resuspended in 2 ml of PBS, 2 ml of freshly prepared nuclear isolation buffer [1.28 M sucrose, 40 mM tris-HCl (pH 7.5), 20 mM MgCl_2_, and 4% Triton X-100] and 6 ml of nuclease-free water on ice for 20 min with frequent mixing. Nuclei were centrifugated at 2500*g* for 15 min. Nuclear pellet was resuspended in 1 ml of RIP buffer [150 mM KCl, 25 mM tris (pH 7.4), 5 mM EDTA, 0.5 mM DTT, 0.5% IGEPAL CA-630, 1× Proteinase inhibitor, and Ribolock RNase inhibitor (100 U/ml)]. Resuspended nuclei were mechanically sheared using a dounce homogenizer with 20 to 30 strokes. Nuclear membrane and debris were spun down at 13,000 rpm for 10 min. Supernatant (50 μl) was set aside as an input. The remaining supernatants were divided into two fractions of 500 μl each [for immunoglobulin G (IgG) control and specific antibody]. One of antibodies including H3K27me3 (Abcam, ab192985), H3K9me3 (Abcam, ab8898), and IgG (Abcam, ab171870) was added to each supernatant and incubated overnight at 4°C with gentle rotation. Pierce Protein A/G Plus Agarose beads (20 μl) were added and incubated for 2 hours at 4°C with gentle rotation. Beads were then pelleted at 2500 rpm for 1 min, and the supernatant was discarded. Beads were washed with 500 μl of RIP buffer for three times, followed by one time in PBS, each time for 5 min with a gentle rocking. Beads were resuspended in 1 ml of TRIzol RNA extraction reagent (Invitrogen, 10296010) to isolate the coprecipitated RNAs according to the manufacturer’s instruction. The cDNA was synthesized by performing the reverse transcription using the HiScript II Q RT SuperMix for qPCR (+gDNA wiper) Kit (Vazyme, R223-01). Subsequently, quantitative PCR was performed using the ChamQ Universal SYBR qPCR Master Mix (Vazyme, Q711-02) with the primers specified in data file S1. The results were analyzed using the GraphPad Prism software (v 9.0).

### Western blot

Cells were rinsed once using DPBS (Thermo Fisher Scientific, 14190235) in the 12-well plates, and four wells of cells were collected together as one sample for further treatment. Each sample was then lysed at room temperature for 30 min using 200 μl of RIPA buffer [140 mM NaCl, 1 mM EDTA (pH 8.0), 1% Triton X-100, 0.1% SDS, 0.1% sodium deoxycholate, and 10 mM tris-HCl (pH 8.0)] containing 2% benzonase (Beyotime, D7121-25KU). The lysate was centrifuged at 12,000 rpm for 2 min. The supernatant was transferred to 1.5-ml tubes, and the protein concentration was measured using the PierceTM BCA Protein Assay Kit (Thermo Fisher Scientific, 23227). After normalizing the protein concentration, the samples were mixed with loading buffer and denatured at 95°C for 10 min. An equal amount of protein was loaded into an SDS–polyacrylamide gel electrophoresis gel (Beyotime, P0468) for electrophoresis followed by polyvinylidene difluoride (PVDF) membrane (Bio-Rad, 1620177) transferring. The Mini-PROTEAN Tetra Cell (Bio-Rad, 1658004) was used in both the electrophoresis and membrane transferring process, and the Power Pac Basic Power Supply (Bio-Rad, 1645050) was used as the power supply equipment. The gel electrophoresis was performed in tris-glycine buffer (0.025 M tris (Trizma base, VETEC, V900483), 0.25 M glycine (Sigma-Aldrich, 50046), and 0.0035 M SDS (Sangon Biotech, A600485) for around 1.5 hours at the voltage of 120 V. The Western blot transfer was conducted in the transfer buffer [0.025 M tris and 0.2 M glycine) containing 10% methanol (Anaqua Global International, MA-1291) for 45 min at the voltage of 200 mA. In addition, Tris Buffered Saline with Tween 20 (TBST) buffer was used to wash the PVDF membrane. The Bio-Rad ChemiDoc Imaging System and Fiji (v 2.14.0) were used for the visualization and quantification of the Western blot signal. The H3K9me3 (diluted 1:2000; Active Motif, 39161), H3K27me3 (diluted 1:1000; Abcam, ab192985), and the Histone 3 primary antibody (diluted 1:1000; Beyotime, AF0009) were used in this assay. The horseradish peroxidase (HRP)–labeled Goat Anti-Mouse IgG (H+L) (diluted 1:3000; Beyotime, A0216) and HRP-labeled Goat Anti-Rabbit IgG (H+L) (diluted 1:3000; Beyotime, A0208) were used as the secondary antibodies. The SuperSignal West Femto Maximum Sensitivity Substrate (Thermo Fisher Scientific, 34094) was used for the chemiluminescence.

### Immunofluorescence microscopy

HEK293T cells grown on the coverslips (18 mm; Nest, 801011) were first transfected with ASOs including ASOXNC23, ASOSETD5-AS1, ASOLINC00641, and ASO LINC00662 for 48 hours to knock down the corresponding gene expression before immunofluorescence (sequence information in data file S1). Cells were rinsed with warm PBS and fixed in 4% paraformaldehyde (Biosharp, BL539A) at room temperature for 30 min. Permeabilization was performed twice with Phosphate-buffered saline with Tween 20 (PBST) containing 0.1% Triton X-100 (Sigma-Aldrich, X100) and 0.01% Tween (Sigma-Aldrich, P9416) for 10 min each time. After blocking with 1% BSA for 30 min, rabbit antibodies against H3K9me3 (diluted 1:500; Abcam, ab8898) and H3K27me3 (diluted 1:500; Abcam, ab192985) were used as the primary antibodies for incubation with permeabilized cells overnight at 4°C. Cells were then washed three times with PBST before incubation with the secondary antibodies. The following procedures, including the usage of secondary antibody and (DAPI), were conducted under darkness. Goat anti-Rabbit IgG HL Alexa Fluor 488 (diluted 1:500; Abcam, ab150077) was used by incubating with the slides at 37°C for 1 hour, and the slides were also counterstained with DAPI (1 μg/ml; Invitrogen, D3571) for 10 min followed by three times washed with PBST before being mounted with Antifade Mountant (ProLong Glass Antifade Mountant, Invitrogen, P36982). Hereafter, the pictures were taken with the laser scanning confocal microscope (Nikon A1HD25, at TBSC, City University of Hong Kong). All images were analyzed with Python (3.11.3) package OpenCV (4.8.0). Briefly, to eliminate the difference of brightness in different images, all images were normalized on the basis of the distribution of the brightness, namely, setting the brightness values of the image background and the brightest point to 0 and 255, respectively. To obtain the individual cell profile, a global threshold was applied to depict individual nucleus, followed by the watershed algorithm (Serge Beucher and Christian Lantuéj workshop on image processing, real-time edge and motion detection, 1979) to separate overlapping and adhering cells. The area with the fluorescence signal above the threshold (77 of 255 in this case) was recognized as positive stained area. By combining all positive areas within one cell divided by the total area of the cell, the term “fluorescence density” could be defined. Then, fluorescence density of multiple cells (H3K27me3: NC group, *n* = 179; ΔSETD5-AS1, *n* = 83; ΔLINC00641, *n* = 110. H3K9me3: NC group, *n* = 53; ΔLINC00662, *n* = 52) for one experiment was collected for comparison. Student’s *t* test was used to assess the significance of difference between different experiments.

### Chrom-seq data analysis

For Chrom-seq, we performed initial quality control of the sequencing data using FastQC (v0.11.8). In our library construction, Read1 had the sequence “NNNNNNNNGGG” at the 5′ end, and Read2 had “NNNNNNNNCCC” at the 3′ end. Subsequently, we trimmed these sequences using fastp (v0.23.1) with the parameters: fastp -A -f 11 -f 0 -t 0 -t 11. The trimmed reads were then aligned to the hg19 (GRCh37.p13) or mm10 (GRCm38.p6) genome using HISAT2 (v2.1.0) with the parameter: hisat2 --no-unal. The aligned reads were converted to BAM files and sorted using SAMtools (v1.11). Replicates BAM files were merged and then converted to BigWig files and normalized for visualization in Integrative Genomics Viewer (IGV, v2.16.1) using deepTools (v3.5.1) with the parameters: bamCoverage -bs 10 –normalize using reads per kilobase per million mapped reads (RPKM). To obtain raw read counts, we quantified the aligned reads using featureCounts (v2.0.1) based on the GENCODE v19 or vM25 annotation. To filter out lowly expressed genes, we screened out genes with the sum of the raw count >18 (i.e., average of three per sample) in three replicates of treated and control groups, and then differential expression analysis was performed using DEseq2 (v1.34.0) with threshold: log_2_-transformed fold change ≥ 0.75 and FDR < 0.05. The volcano plots for visualization of differential expression analysis results were generated using R packages ggplot2 (v3.4.2), RColorBrewer (v1.1-3), and ggrepel (v0.9.3). For RNA-seq data analysis, we conducted the same pipeline as the Chrom-seq.

To determine the proportion of Chrom-seq reads in features, we used QualiMap (v2.2.1) with the parameters: qualimap rnaseq -a uniquely-mapped-reads -p strand-specific-forward -pe -s. Donut chart was generated using R package webr (v0.1.5). In the three-dimensional (3D) pie chart, the number of protein coding and ncRNAs were screened by gene type under the threshold of log_2_-transformed fold change ≥ 0.75 and FDR < 0.05. The 3D pie charts were generated using R package plotrix (v3.8-2). To compare RNAs enriched in reader module with the overall expression level in the cellular transcriptome, raw counts of untreated HEK293T cells quantified by featureCounts were converted to fragments per kilobase of transcript per million mapped reads (FPKM) values. Box plots depicting the expression levels of RNA enriched or nonenriched in reader modules were generated using R packages ggplot2, tidyverse (2.0.0), and reshape2 (v1.4.4). For correlation analysis between Chrom-seq component expression and gene expression in cells, raw counts of untreated HEK293T and CBX7 module experimental groups were converted to FPKM values and log_2_-transformed. Normality tests were performed, and scatter plot was generated using R packages ggplot2, ggpubr (v0.6.0), ggExtra (v0.10.0), and Spearman’s ρ and *P* value were calculated using ggpubr stat_cor function. To assess changes in genes near histone modification peaks upon depletion of lncRNA, genes adjacent to histone modification peaks were obtained using bedtools (v2.30.0) closest, and gene expression levels are normalized by DESeq’ RLE (relative log expression). To ensure an adequate number of reads in Chrom-seq sequencing libraries, we used sequencing libraries with varying reads to identify ncRNAs. Line chart was generated using Python (v3.8.0) package matplotlib (v3.4.2).

To compare the H3K4me3-associated RNAs identified by Chrom-seq and PIRCh-seq, we downloaded H3K4me3 PIRCh-seq data of H9 cells from the Gene Expression Omnibus (GEO) database (GSE119006). The raw reads were aligned to the hg19 genome using HISAT2 and quantified using featureCounts based on the GENCODE v19 annotation. To filter out lowly expressed genes, we screening out genes with the sum of the raw count >12 (i.e., average of three per sample) in two replicates of treated and control groups, and differential expression analysis was performed using DESeq2 with threshold: log_2_-transformed fold change > 0 and *P* value < 0.05. Bar plots depicting ncRNA types based on GENCODE annotation were generated using R packages ggplot2 and tidyverse. To compare the abundance of ncRNAs identified by PIRCh-seq and Chrom-seq in H9 and HEK293T cells, raw counts of untreated H9 and HEK293T cells quantified by featureCounts were converted to FPKM values. The expression levels of H3K4me3 PIRCh-seq and TAF3 Chrom-seq enriched or nonenriched RNAs were extracted from FPKM converted counts.

To classify chromatin-associated ncRNAs, we merged the ncRNAs in HEK293T cells identified by CBX1, CBX7, and TAF3 readers in Chrom-seq into a union set and transformed into a 1618 by 3 matrix. Rows in the matrix represent ncRNAs identified by Chrom-seq, columns in the matrix represent histone modifications represented by different readers, and each element in the matrix represents the log_2_ fold enrichment of the corresponding ncRNA on a specific histone modification. Subsequently, t-distributed stochastic neighbor embedding (t-SNE) dimensionality reduction and K-means clustering analysis were performed to divide ncRNAs into three clusters. For each cluster, the relative contribution of chromatin-associated RNAs to each histone modification was evaluated, defining the clustering status as repressive, bivalent, and active. Figure 3N and fig. S5C were generated using Python package seaborn (v0.11.2). This section of the analysis refers to PIRCh-seq (*13*).

### ChIP-seq data analysis

For ChIP-seq, the preliminary quality control of the sequencing data was performed using FastQC. Cutadapt (v3.3) was used to remove adapter from the sequencing reads. Subsequently, the trimmed reads were then aligned to the hg19 genome using Bowtie2 (v2.4.2) with the parameter: bowtie2 --no-unal. PCR duplicates were removed using GATK MarkDuplicates (v4.1.9.0) with the parameters: gatk MarkDuplicates --REMOVE_DUPLICATES true. The replicates BAM files were merged, and peak calling was performed using MACS2 (v2.2.7.1) with the parameters: macs2 callpeak -f BAMPE -g hs. The BedGraph files were generated by the peak calling result and converted to BigWig files using bedGraphToBigWig for visualization in IGV. Then, IP divided by input to obtain the combined tracks by using the IGV tools (combine data tracks).

To demonstrate changes in histone modification peaks upon depletion of lncRNA, the BAM files of histone and depleted lncRNA were converted to BigWig files for visualization using deepTools with the following parameters: bamCompare --binSize 10 --skipZeroOverZero --skipNAs -bl hg19-blacklist.v2.bed --centerReads. Subsequently, it is visualized using deepTools plotHeatmap. To compare the RNAs identified by Chrom-seq with the ChIP-seq signals of different histone modifications for other genes, we downloaded the ChIP-seq data of H3K4me3 in HEK293T cells from the GEO database (GSM1249885). Under the threshold of log_2_ fold change ≥ 0.75 and FDR < 0.05, enriched and nonenriched RNAs were extracted from the results of differential expression analysis, and the gene body and 1000 upstream and downstream regions of these RNAs were used as the regions for signal distribution. Subsequently, the BAM files of histone ChIP-seq were converted to BigWig files using deepTools with the parameter bamCoverage -bs 10 and visualized using deepTools plotProfile.

To obtain chromatin modification peaks in selected genomic regions, we downloaded H3K4me3 ChIP-seq (with input) data from HEK293 cells from the GEO database (GSE35583). For the Pearson correlation heatmap analysis, we divided the entire hg19 genome into 1-kb intervals using bedtools makewindows and removed the portions overlapping with satellite repeat regions from the intervals. To reduce artifacts, we further removed the ENCODE blacklist regions of the hg19 genome and portions with mappability scores of <0.5 within the genomic intervals, and the mappability scores were calculated using the GEMTools (V1.7.1) software. Next, we merged the peaks of the histone modifications H3K4me3, H3K9me3, and H3K27me3 to obtain genomic intervals containing these peaks, which were used to calculate the correlation between the treatment with the CBX7 module and the antibody signals of histone modifications. Pearson correlation scores were generated by deepTools plotCorrelation. This section of the analysis referred to ChromID (*19*).

### Statistical analysis

For the RT-qPCR data in bar plots, *P* values were calculated by Student’s *t* test in GraphPad Prism (v 9.0). For the data in Figs. 1J and 2I, and *P* values were calculated by the Wilcoxon signed-rank test in R. For the data in Fig. 3G and figs. S1 (B and F), S2F, and S4H, *P* values were calculated by the Mann-Whitney *U* test in R. For the data in figs. S3D and S4F, *P* values were calculated by Student’s *t* test in Python. All statistical tests were two-tailed.

## References

[1] J. Yan, S. A. Chen, A. Local, T. Liu, Y. Qiu, K. M. Dorighi, S. Preissl, C. M. Rivera, C. Wang, Z. Ye, K. Ge, M. Hu, J. Wysocka, B. Ren, Histone H3 lysine 4 monomethylation modulates long-range chromatin interactions at enhancers. Cell Res. 28, 387 (2018). 29497152 10.1038/cr.2018.18 PMC5835777

[2] K. K. Sinha, S. Bilokapic, Y. Du, D. Malik, M. Halic, Histone modifications regulate pioneer transcription factor cooperativity. Nature 619, 378–384 (2023). 37225990 10.1038/s41586-023-06112-6 PMC10338341

[3] H. Mokrani, O. Sharaf el Dein, Z. Mansuroglu, E. Bonnefoy, Binding of YY1 to the proximal region of the murine beta interferon promoter is essential to allow CBP recruitment and K8H4/K14H3 acetylation on the promoter region after virus infection. Mol. Cell. Biol. 26, 8551–8561 (2006). 16954376 10.1128/MCB.00420-06 PMC1636788

[4] J. C. Chuang, P. A. Jones, Epigenetics and microRNAs. Pediatr. Res. 61, 24R–29R (2007). 10.1203/pdr.0b013e3180457684 17413852

[5] Y. Yu, Q. Chen, X. Zhang, J. Yang, K. Lin, C. Ji, A. Xu, L. Yang, L. Miao, Long noncoding RNA ANRIL promotes the malignant progression of cholangiocarcinoma by epigenetically repressing ERRFI1 expression. Cancer Sci. 111, 2297–2309 (2020). 32378752 10.1111/cas.14447 PMC7385372

[6] H. Fang, G. Bonora, J. P. Lewandowski, J. Thakur, G. N. Filippova, S. Henikoff, J. Shendure, Z. Duan, J. L. Rinn, X. Deng, W. S. Noble, C. M. Disteche, Trans- and cis-acting effects of Firre on epigenetic features of the inactive X chromosome. Nat. Commun. 11, 6053 (2020). 33247132 10.1038/s41467-020-19879-3 PMC7695720

[7] N. Singh, V. R. Ramnarine, J. H. Song, R. Pandey, S. K. R. Padi, M. Nouri, V. Olive, M. Kobelev, K. Okumura, D. McCarthy, M. M. Hanna, P. Mukherjee, B. Sun, B. R. Lee, J. B. Parker, D. Chakravarti, N. A. Warfel, M. Zhou, J. J. Bearss, E. A. Gibb, M. Alshalalfa, R. J. Karnes, E. J. Small, R. Aggarwal, F. Feng, Y. Wang, R. Buttyan, A. Zoubeidi, M. Rubin, M. Gleave, F. J. Slack, E. Davicioni, H. Beltran, C. Collins, A. S. Kraft, The long noncoding RNA H19 regulates tumor plasticity in neuroendocrine prostate cancer. Nat. Commun. 12, 7349 (2021). 34934057 10.1038/s41467-021-26901-9 PMC8692330

[8] J. Hu, Y. Shan, J. Ma, Y. Pan, H. Zhou, L. Jiang, L. Jia, LncRNA ST3Gal6-AS1/ST3Gal6 axis mediates colorectal cancer progression by regulating α-2,3 sialylationviaPI3K/Akt signaling. Int. J. Cancer 145, 450–460 (2019). 30613961 10.1002/ijc.32103

[9] I. V. Bure, M. V. Nemtsova, E. B. Kuznetsova, Histone modifications and non-coding RNAs: Mutual epigenetic regulation and role in pathogenesis. Int. J. Mol. Sci. 23, (2022). 10.3390/ijms23105801 PMC9146199 35628612

[10] L. Zhao, J. Wang, Y. Li, T. Song, Y. Wu, S. Fang, D. Bu, H. Li, L. Sun, D. Pei, Y. Zheng, J. Huang, M. Xu, R. Chen, Y. Zhao, S. He, NONCODEV6: An updated database dedicated to long non-coding RNA annotation in both animals and plants. Nucleic Acids Res. 49, D165–D171 (2021). 33196801 10.1093/nar/gkaa1046 PMC7779048

[11] T. Mondal, S. Subhash, C. Kanduri, Chromatin RNA immunoprecipitation (ChRIP). Methods Mol. Biol. 1689, 65–76 (2018). 29027165 10.1007/978-1-4939-7380-4_6

[12] B. L. Kidder, CARIP-Seq and ChIP-Seq: methods to identify chromatin-associated RNAs and Protein-DNA interactions in embryonic stem cells. J. Vis. Exp. (2018). 10.3791/57481 PMC6101426 29889205

[13] J. Fang, Q. Ma, C. Chu, B. Huang, L. Li, P. Cai, P. J. Batista, K. E. M. Tolentino, J. Xu, R. Li, P. Du, K. Qu, H. Y. Chang, PIRCh-seq: Functional classification of non-coding RNAs associated with distinct histone modifications. Genome Biol. 20, 292 (2019). 31862000 10.1186/s13059-019-1880-3 PMC6924075

[14] N. Khyzha, S. Henikoff, K. Ahmad, Profiling RNA at chromatin targets in situ by antibody-targeted tagmentation. Nat. Methods 19, 1383–1392 (2022). 36192462 10.1038/s41592-022-01618-9 PMC9636022

[15] S. Subhash, K. Mishra, V. S. Akhade, M. Kanduri, T. Mondal, C. Kanduri, H3K4me2 and WDR5 enriched chromatin interacting long non-coding RNAs maintain transcriptionally competent chromatin at divergent transcriptional units. Nucleic Acids Res. 46, 9384–9400 (2018). 30010961 10.1093/nar/gky635 PMC6182144

[16] G. Kungulovski, I. Kycia, R. Tamas, R. Z. Jurkowska, S. Kudithipudi, C. Henry, R. Reinhardt, P. Labhart, A. Jeltsch, Application of histone modification-specific interaction domains as an alternative to antibodies. Genome Res. 24, 1842–1853 (2014). 25301795 10.1101/gr.170985.113 PMC4216925

[17] F. M. Fazal, S. Han, K. R. Parker, P. Kaewsapsak, J. Xu, A. N. Boettiger, H. Y. Chang, A. Y. Ting, Atlas of subcellular RNA localization revealed by APEX-Seq. Cell 178, 473–490.e26 (2019). 31230715 10.1016/j.cell.2019.05.027 PMC6786773

[18] Y. Zhou, G. Wang, P. Wang, Z. Li, T. Yue, J. Wang, P. Zou, Expanding APEX2 substrates for proximity-dependent labeling of nucleic acids and proteins in living cells. Angew. Chem. Int. Ed. Engl. 58, 11763–11767 (2019). 31240809 10.1002/anie.201905949

[19] R. Villasenor, R. Pfaendler, C. Ambrosi, S. Butz, S. Giuliani, E. Bryan, T. W. Sheahan, A. L. Gable, N. Schmolka, M. Manzo, J. Wirz, C. Feller, C. von Mering, R. Aebersold, P. Voigt, T. Baubec, ChromID identifies the protein interactome at chromatin marks. Nat. Biotechnol. 38, 728–736 (2020). 32123383 10.1038/s41587-020-0434-2 PMC7289633

[20] M. E. Tanenbaum, L. A. Gilbert, L. S. Qi, J. S. Weissman, R. D. Vale, A protein-tagging system for signal amplification in gene expression and fluorescence imaging. Cell 159, 635–646 (2014). 25307933 10.1016/j.cell.2014.09.039 PMC4252608

[21] Z. Du, X. Shi, A. Guan, lncRNA H19 facilitates the proliferation and differentiation of human dental pulp stem cells via EZH2-dependent LATS1 methylation. Mol Ther Nucleic Acids 25, 116–126 (2021). 34401209 10.1016/j.omtn.2021.04.017 PMC8339349

[22] Q. Wang, L. Liu, S. Zhang, Y. Ming, S. Liu, K. Cheng, Y. Zhao, Long noncoding RNA NEAT1 suppresses hepatocyte proliferation in fulminant hepatic failure through increased recruitment of EZH2 to the LATS2 promoter region and promotion of H3K27me3 methylation. Exp. Mol. Med. 52, 461–472 (2020). 32157157 10.1038/s12276-020-0387-z PMC7156754

[23] D. Qu, W. W. Sun, L. Li, L. Ma, L. Sun, X. Jin, T. Li, W. Hou, J. H. Wang, Long noncoding RNA MALAT1 releases epigenetic silencing of HIV-1 replication by displacing the polycomb repressive complex 2 from binding to the LTR promoter. Nucleic Acids Res. 47, 3013–3027 (2019). 30788509 10.1093/nar/gkz117 PMC6451131

[24] M. Shao, G. Chen, F. Lv, Y. Liu, H. Tian, R. Tao, R. Jiang, W. Zhang, C. Zhuo, LncRNA TINCR attenuates cardiac hypertrophy by epigenetically silencing CaMKII. Oncotarget 8, 47565–47573 (2017). 28548932 10.18632/oncotarget.17735 PMC5564587

[25] Y. Tai, Y. Ji, F. Liu, Y. Zang, D. Xu, S. Ma, L. Qin, J. Ma, Long noncoding RNA SOX2-OT facilitates laryngeal squamous cell carcinoma development by epigenetically inhibiting PTEN via methyltransferase EZH2. IUBMB Life 71, 1230–1239 (2019). 30811870 10.1002/iub.2026

[26] S. Guil, M. Soler, A. Portela, J. Carrere, E. Fonalleras, A. Gomez, A. Villanueva, M. Esteller, Intronic RNAs mediate EZH2 regulation of epigenetic targets. Nat. Struct. Mol. Biol. 19, 664–670 (2012). 22659877 10.1038/nsmb.2315

[27] J. Zhao, T. K. Ohsumi, J. T. Kung, Y. Ogawa, D. J. Grau, K. Sarma, J. J. Song, R. E. Kingston, M. Borowsky, J. T. Lee, Genome-wide identification of polycomb-associated RNAs by RIP-seq. Mol. Cell 40, 939–953 (2010). 21172659 10.1016/j.molcel.2010.12.011 PMC3021903

[28] A. R. Karapetyan, C. Buiting, R. A. Kuiper, M. W. Coolen, Regulatory roles for long ncRNA and mRNA. Cancers 5, 462–490 (2013). 24216986 10.3390/cancers5020462 PMC3730338

[29] R. Margueron, D. Reinberg, The Polycomb complex PRC2 and its mark in life. Nature 469, 343–349 (2011). 21248841 10.1038/nature09784 PMC3760771

[30] Y. Huang, J. Wang, Y. Zhao, H. Wang, T. Liu, Y. Li, T. Cui, W. Li, Y. Feng, J. Luo, J. Gong, L. Ning, Y. Zhang, D. Wang, Y. Zhang, cncRNAdb: A manually curated resource of experimentally supported RNAs with both protein-coding and noncoding function. Nucleic Acids Res. 49, D65–D70 (2021). 33010163 10.1093/nar/gkaa791 PMC7778915

[31] H. Sunwoo, J. Y. Wu, J. T. Lee, The Xist RNA-PRC2 complex at 20-nm resolution reveals a low Xist stoichiometry and suggests a hit-and-run mechanism in mouse cells. Proc. Natl. Acad. Sci. U. S. A. 112, E4216–E4225 (2015). 26195790 10.1073/pnas.1503690112 PMC4534268

[32] P. Monnier, C. Martinet, J. Pontis, I. Stancheva, S. Ait-Si-Ali, L. Dandolo, H19 lncRNA controls gene expression of the Imprinted Gene Network by recruiting MBD1. Proc. Natl. Acad. Sci. U. S. A. 110, 20693–20698 (2013). 24297921 10.1073/pnas.1310201110 PMC3870736

[33] X. Chen, L. He, Y. Zhao, Y. Li, S. Zhang, K. Sun, K. So, F. Chen, L. Zhou, L. Lu, L. Wang, X. Zhu, X. Bao, M. A. Esteban, S. Nakagawa, K. V. Prasanth, Z. Wu, H. Sun, H. Wang, Malat1 regulates myogenic differentiation and muscle regeneration through modulating MyoD transcriptional activity. Cell Discov. 3, 17002 (2017). 28326190 10.1038/celldisc.2017.2 PMC5348715

[34] D. C. Schultz, K. Ayyanathan, D. Negorev, G. G. Maul, F. J. Rauscher III, SETDB1: A novel KAP-1-associated histone H3, lysine 9-specific methyltransferase that contributes to HP1-mediated silencing of euchromatic genes by KRAB zinc-finger proteins. Genes Dev. 16, 919–932 (2002). 11959841 10.1101/gad.973302 PMC152359

[35] S. Rea, F. Eisenhaber, D. O'Carroll, B. D. Strahl, Z. W. Sun, M. Schmid, S. Opravil, K. Mechtler, C. P. Ponting, C. D. Allis, T. Jenuwein, Regulation of chromatin structure by site-specific histone H3 methyltransferases. Nature 406, 593–599 (2000). 10949293 10.1038/35020506

[36] A. Frapporti, C. Miro Pina, O. Arnaiz, D. Holoch, T. Kawaguchi, A. Humbert, E. Eleftheriou, B. Lombard, D. Loew, L. Sperling, K. Guitot, R. Margueron, S. Duharcourt, The Polycomb protein Ezl1 mediates H3K9 and H3K27 methylation to repress transposable elements in Paramecium. Nat. Commun. 10, 2710 (2019). 31221974 10.1038/s41467-019-10648-5 PMC6586856

[37] S. Bilodeau, M. H. Kagey, G. M. Frampton, P. B. Rahl, R. A. Young, SetDB1 contributes to repression of genes encoding developmental regulators and maintenance of ES cell state. Genes Dev. 23, 2484–2489 (2009). 19884255 10.1101/gad.1837309 PMC2779743

[38] O. Alder, F. Lavial, A. Helness, E. Brookes, S. Pinho, A. Chandrashekran, P. Arnaud, A. Pombo, L. O'Neill, V. Azuara, Ring1B and Suv39h1 delineate distinct chromatin states at bivalent genes during early mouse lineage commitment. Development 137, 2483–2492 (2010). 20573702 10.1242/dev.048363 PMC2927698

[39] E. Bernstein, E. M. Duncan, O. Masui, J. Gil, E. Heard, C. D. Allis, Mouse polycomb proteins bind differentially to methylated histone H3 and RNA and are enriched in facultative heterochromatin. Mol. Cell. Biol. 26, 2560–2569 (2006). 16537902 10.1128/MCB.26.7.2560-2569.2006 PMC1430336

[40] A. Kapusta, A. Suh, C. Feschotte, Dynamics of genome size evolution in birds and mammals. Proc. Natl. Acad. Sci. U. S. A. 114, E1460–E1469 (2017). 28179571 10.1073/pnas.1616702114 PMC5338432

[41] S. G. Landt, G. K. Marinov, A. Kundaje, P. Kheradpour, F. Pauli, S. Batzoglou, B. E. Bernstein, P. Bickel, J. B. Brown, P. Cayting, Y. Chen, G. DeSalvo, C. Epstein, K. I. Fisher-Aylor, G. Euskirchen, M. Gerstein, J. Gertz, A. J. Hartemink, M. M. Hoffman, V. R. Iyer, Y. L. Jung, S. Karmakar, M. Kellis, P. V. Kharchenko, Q. Li, T. Liu, X. S. Liu, L. Ma, A. Milosavljevic, R. M. Myers, P. J. Park, M. J. Pazin, M. D. Perry, D. Raha, T. E. Reddy, J. Rozowsky, N. Shoresh, A. Sidow, M. Slattery, J. A. Stamatoyannopoulos, M. Y. Tolstorukov, K. P. White, S. Xi, P. J. Farnham, J. D. Lieb, B. J. Wold, M. Snyder, ChIP-seq guidelines and practices of the ENCODE and modENCODE consortia. Genome Res. 22, 1813–1831 (2012). 22955991 10.1101/gr.136184.111 PMC3431496

[42] M. Busby, C. Xue, C. Li, Y. Farjoun, E. Gienger, I. Yofe, A. Gladden, C. B. Epstein, E. M. Cornett, S. B. Rothbart, C. Nusbaum, A. Goren, Systematic comparison of monoclonal versus polyclonal antibodies for mapping histone modifications by ChIP-seq. Epigenetics Chromatin 9, 49 (2016). 27826357 10.1186/s13072-016-0100-6 PMC5097419

[43] P. C. He, C. He, m(6) A RNA methylation: From mechanisms to therapeutic potential. EMBO J. 40, e105977 (2021). 33470439 10.15252/embj.2020105977 PMC7849164

[44] P. P. Vaidyanathan, I. AlSadhan, D. K. Merriman, H. M. Al-Hashimi, D. Herschlag, Pseudouridine and N(6)-methyladenosine modifications weaken PUF protein/RNA interactions. RNA 23, 611–618 (2017). 28138061 10.1261/rna.060053.116 PMC5393172

[45] M. Hagemann-Jensen, C. Ziegenhain, P. Chen, D. Ramskold, G. J. Hendriks, A. J. M. Larsson, O. R. Faridani, R. Sandberg, Single-cell RNA counting at allele and isoform resolution using Smart-seq3. Nat. Biotechnol. 38, 708–714 (2020). 32518404 10.1038/s41587-020-0497-0

[46] J. L. Rinn, M. Kertesz, J. K. Wang, S. L. Squazzo, X. Xu, S. A. Brugmann, L. H. Goodnough, J. A. Helms, P. J. Farnham, E. Segal, H. Y. Chang, Functional demarcation of active and silent chromatin domains in human HOX loci by noncoding RNAs. Cell 129, 1311–1323 (2007). 17604720 10.1016/j.cell.2007.05.022 PMC2084369

