# Supplementary material for: Chrom-seq identifies RNAs at chromatin marks
Source: Sci Adv. 2024 Jul 31;10(31):eadn1397. doi: 10.1126/sciadv.adn1397 (PMC11290522; doi:10.1126/sciadv.adn1397)
Supplement: Supplementary file 1 — Figs. S1 to S5 Legends for data S1 to S6 [file sciadv.adn1397_sm.pdf]

Science Advances
AAAS

Supplementary Materials for

Chrom-seq identifies RNAs at chromatin marks

Ligang Fan *et al.*

Corresponding author: Jian Yan, jian.yan@cityu.edu.hk

Sci. Adv. **10**, eadn1397 (2024)
DOI: 10.1126/sciadv.adn1397

The PDF file includes:

Figs. S1 to S5
Legends for data S1 to S6

Other Supplementary Material for this manuscript includes the following:

Data S1 to S6

Fan et al. Supplementary fig. S1

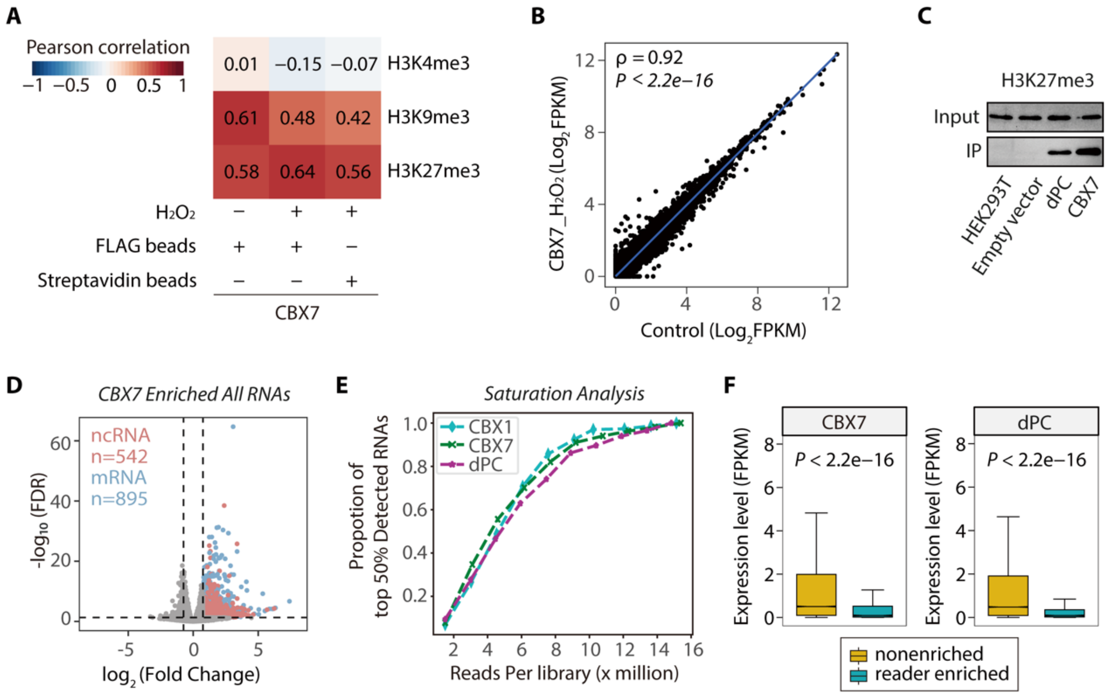

Figure S1: RNAs enriched by CBX7 and dPC readers.

A) Pearson correlation score of chromatin modifications and CBX7 at selected genomic intervals. B) Scatter plot shows transcriptome profile (FPKM) of cells transfected with CBX7 reader correlates with the profile of untreated HEK293T cells (Spearman's ρ=0.92, $P < 2.2\times10^{-16}$), n=3 independent replicates. C) Western blot of H3K27me3 in input and streptavidin-enriched samples. Proteins were collected from HEK293T cells, or cells transfected with empty vector, dPC or CBX7 reader. D) Volcano plot of all RNAs associated with H3K27me3 modification identified by CBX7 Chrom-seq in HEK293T cells. The number of significantly enriched coding RNAs labeled in blue is 895, and the number of enriched noncoding RNAs labeled in red is 542. The dashed lines indicate thresholds for significant enrichment, i.e., $\log_2$-transformed fold change ≥ 0.75, FDR < 0.05, n=3 independent replicates. E) Line chart shows the proportion (y-axis) of the top 50% RNAs significantly captured by Chrom-seq under different levels subsampling of sequencing reads (x-axis). F) Boxplots show the expression level (FPKM) of RNAs enriched or not enriched by CBX7 (left) and dPC (right) reader. Mann–Whitney U test was performed, $P < 2.2\times10^{-16}$. n=3 independent replicates. Outliers are omitted in the boxplots.

Fan et al. Supplementary fig. S2

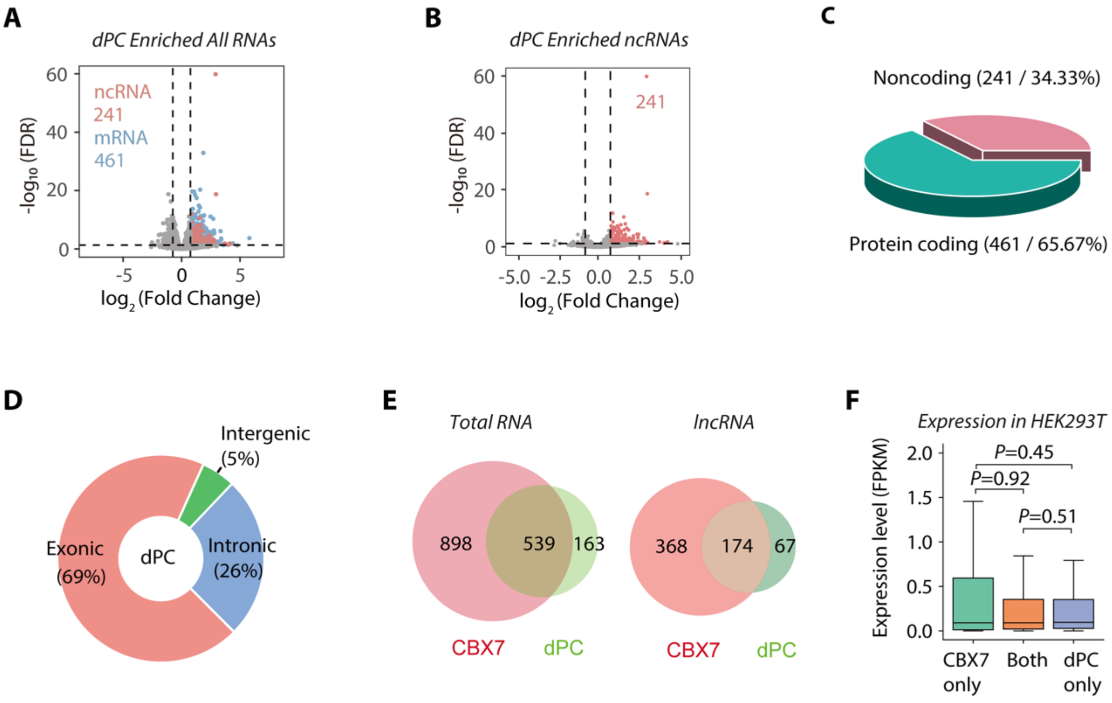

Figure S2: RNAs enriched by dPC reader.

A and B) Volcano plot of RNAs associated with H3K27me3 modification identified by dPC Chrom-seq targeting H3K27me3 in HEK293T cells. The number of significantly enriched coding RNAs labeled in blue is 461, and the number of enriched noncoding RNAs labeled in red is 241. The dashed lines indicate thresholds for significant enrichment, i.e., $\log_2$-transformed fold change ≥ 0.75, FDR < 0.05, n=3 independent replicates. C) 3D pie chart shows the number and percentage of coding and noncoding genes identified in dPC Chrom-seq. D) Donut chart shows the proportion of Chrom-seq captured reads using dPC reader, aligned to feature regions classified as exonic, intronic and intergenic. E) Venn diagram shows comparison of H3K27me3 associated total (left) and ncRNAs (right) found by CBX7 and dPC. F) Box plots show the expression levels of total RNAs enriched by CBX7 and dPC in HEK293T cells, corresponding to panel (E). Mann–Whitney U test is performed. Outliers are omitted.

Fan et al. Supplementary fig. S3

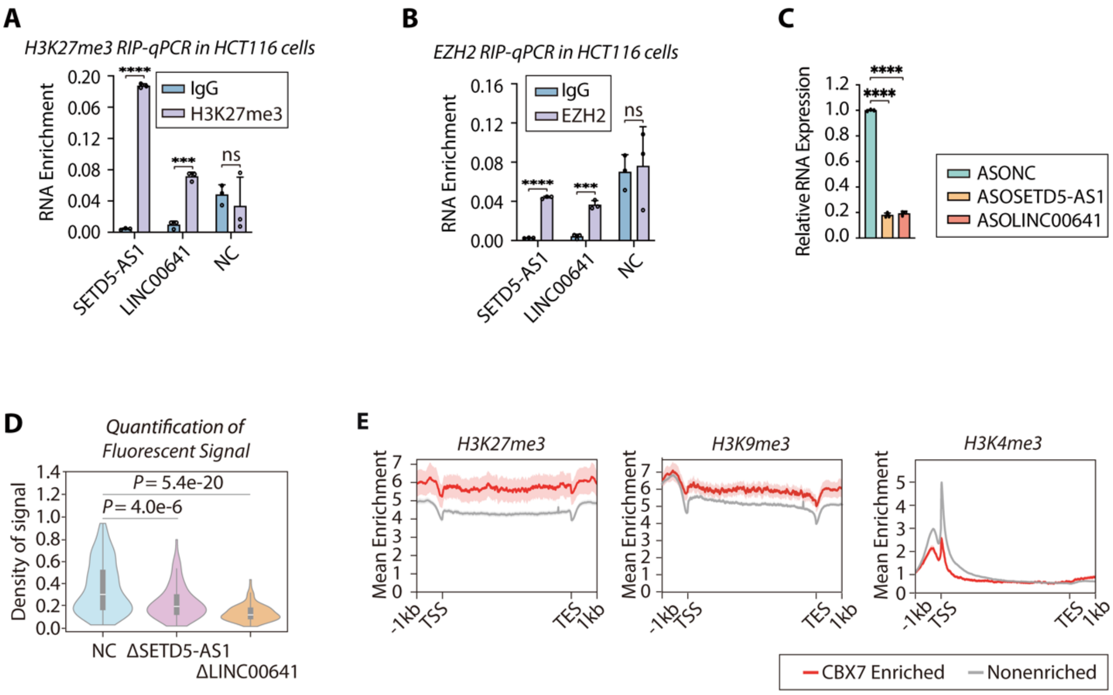

Figure S3: Validation the RNAs associated with H3K27me3 modification identified by Chrom-seq targeting H3K27me3.

A and B) RIP-qPCR validation of candidate genes in HCT116 cells. RNAs were enriched using H3K27me3 (A) and EZH2 (B) antibodies, respectively. IgG was used as control. NC, negative control. Statistic *P* values by two-tailed Student's *t* test are shown; ****P* < 0.001, *****P* < 0.0001. Three independent biological replicates were performed. C) Validation of knock down efficiency of lncRNA SETD5-AS1 and LINC00641 in HEK293T cells. RNAs were isolated from the cell depleted of SETD5-AS1 (ΔSETD5-AS1) and LINC00641 (ΔLINC00641) using ASOs. NC, negative control ASO that does not target any sequence in human transcriptome. Statistic *P* values by two-tailed Student's *t* test are shown; *****P* < 0.0001. Three independent biological replicates were performed. D) Quantification fluorescent signal for Fig. 1H. All images were analyzed with PYTHON (3.11.3) package OpenCV (4.8.0). The number of cells analyzed was 179 in NC, 83 in ΔSETD5-AS1 and 110 in ΔLINC00641, respectively. Two tailed Student's *t* test was used to assess the significance of difference between different experiments. E) The profile plot shows the ChIP signals of H3K27me3, H3K9me3 and H3K4me3 surrounding the 1-kb upstream and downstream

regions around the gene bodies (between transcription starting site, i.e., TSS and transcription termination site, i.e., TES). The RNAs enriched by CBX7 Chrom-seq are labeled in red, and non-enriched genes are labeled in gray. ChIP signals are combined from three replicates of H3K27me3, H3K9me3 and one experiment of H3K4me3. H3K4me3 ChIP data is from GEO database (GSM1249885). Shaded areas indicate standard error.

Fan et al. Supplementary fig. S4

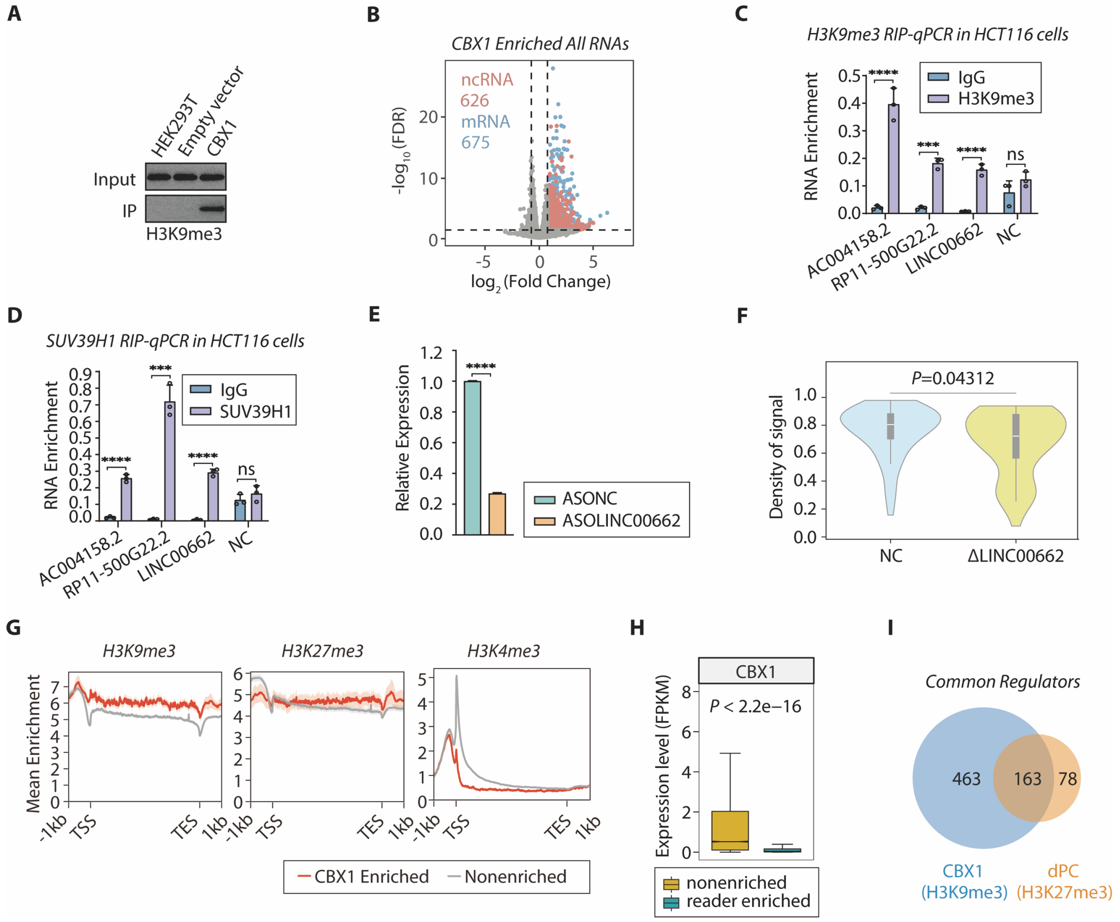

Figure S4: RNAs enriched by CBX1 reader.

A) Western blot of H3K9me3 in input and streptavidin-enriched samples. Proteins were collected from HEK293T cells, or cells transfected with empty vector or CBX1 reader. B) Volcano plot of all RNAs associated with H3K9me3 modification identified by CBX1 Chrom-seq in HEK293T cells. The number of significantly enriched coding RNAs labeled in blue is 675, and the number of enriched noncoding RNAs labeled in red is 626. The dashed lines indicate significance thresholds for significant enrichment, i.e., $\log_2$-transformed fold change ≥ 0.75, FDR < 0.05, n=3 independent replicates. C and D) RIP-qPCR validation of candidate genes in HCT116 cells. RNAs were enriched using H3K9me3 (C) and SUV39H1 (D) antibodies, respectively. IgG was used as control. NC, negative control. Statistic *P* values by two-tailed Student's *t* test are shown; ****P* < 0.001, *****P* < 0.0001. Three independent biological replicates were performed. E) Validation of knocking down efficiency of LINC00662 in HEK293T cells. NC,

negative control ASO that does not target any sequence. Statistic *P* values by two-tailed Student's *t* test are shown; ****$P < 0.0001$. Three independent biological replicates were performed. F) Quantification fluorescent signal for figure 2G. All images were analyzed with PYTHON (3.11.3) package OpenCV (4.8.0). The number of cells analyzed was 53 in NC and 52 in ΔLINC00662, respectively. Statistic *P* value by two-tailed Student's *t* test is shown. G) The profile plot shows the ChIP signals of H3K9me3, H3K27me3 and H3K4me3 surrounding the 1-kb upstream and downstream regions around the gene bodies (between TSS and TES). The RNAs enriched by CBX1 Chrom-seq are labeled in red, and non-enriched RNAs are labeled in gray. ChIP signals are combined from three replicates of H3K9me3 and one replicate of H3K4me3. H3K4me3 ChIP data is from GEO database (GSM1249885). Shaded areas indicate standard error. H) Boxplot shows the expression level (FPKM) of RNAs enriched or nonenriched by CBX1 reader in HEK293T cells. Mann–Whitney U test was conducted, $P < 2.2\times10^{-16}$. The outliers are omitted in the boxplot. I) Venn diagram shows common regulators of H3K27me3 and H3K9me3.

Fan et al. Supplementary fig. S5

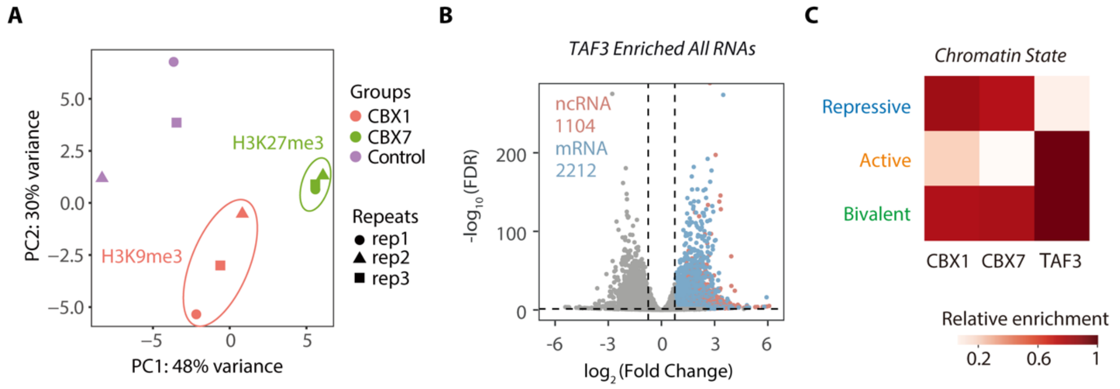

Figure S5: RNAs enriched by TAF3 reader.

A) Principal component analysis shows the clustering of Chrom-seq results from replicative experiments using different reader modules and control. B) Volcano plot of all RNAs associated with H3K4me3 modification identified by TAF3 Chrom-seq in HEK293T cells. The number of significantly enriched coding RNAs labeled in blue is 2,212, and the number of enriched noncoding RNAs labeled in red is 1,104. The dashed lines indicate thresholds for significant enrichment, i.e., $\log_2$-transformed fold change ≥ 0.75, FDR < 0.05, n=3 independent replicates. C) Functional classification of chromatin-RNA association patterns. Color bar indicates the enrichment of chromatin-associated ncRNAs in each chromatin state.

Supplementary Data Files:

Data S1. Sequences of amino acid and oligonucleotide used in this study.

Data S2. RNAs enriched by CBX7 and dPC reader module.

Data S3. Quality matrix of Chrom-seq data in different cells.

Data S4. RNAs enriched by CBX1 and CBX1_W42A reader module.

Data S5. RNAs enriched by TAF3 reader module in different cells.

Data S6. Functional classification of chromatin-associated ncRNAs.
